# TFEB overexpression alleviates autophagy-lysosomal deficits caused by progranulin insufficiency

**DOI:** 10.1038/s41598-025-12268-0

**Published:** 2025-07-19

**Authors:** Wren O. Nader, Kaylan S. Brown, Nicholas R. Boyle, Azariah K. Kaplelach, Shaimaa M. Abdelaziz, Skylar E. Davis, Qays Aljabi, Ahmad R. Hakim, Amelia G. Davidson, Giacynta A. Vollmer, Leah C. Wright, J. Bailey Echols, Joelle Saad, Nicholas S. Pena, Andrew E. Arrant

**Affiliations:** Killion Center for Neurodegeneration and Experimental Therapeutics, Alzheimer’s Disease Center, Evelyn F. McKnight Brain Institute, Department of Neurology, University of Alabama at Birmingham, Birmingham, AL USA

**Keywords:** Progranulin, TFEB, Lysosomes, Autophagy, Dementia, Neurodegeneration, Autophagy, Lysosomes

## Abstract

Progranulin is a pro-protein that is necessary for maintaining lysosomal function. Loss-of-function progranulin (*GRN*) mutations are a dominant cause of frontotemporal dementia (FTD). Brains of people with FTD due to *GRN* mutations accumulate lysosomal storage material and exhibit increased expression of lysosomal transcripts, which may be driven by TFEB and related transcription factors. While this may be a compensatory response to lysosomal impairment, overproduction of lysosomal proteins may also contribute to FTD pathogenesis. To investigate how TFEB may contribute to disease in people with *GRN* mutations, we analyzed the effects of TFEB overexpression in progranulin-insufficient cells and mice. We generated *GRN* knockout HEK-293 cells (*GRN* KO cells), which exhibited increased nuclear localization of TFEB and expression of lysosomal transcripts, but impaired autophagy. TFEB overexpression in *GRN* KO cells further increased lysosomal transcripts and partially normalized autophagy. We next injected an AAV vector expressing mouse *Tfeb* (AAV-TFEB) into the thalamus of *Grn*^*–/–*^ mice, which accumulates lysosomal storage material. AAV-TFEB increased lysosomal transcripts and reduced immunoreactivity for SCMAS, a marker of lysosomal storage material, in *Grn*^*–/–*^ thalamus. These data show that TFEB activity alleviates some autophagy-lysosomal deficits caused by progranulin insufficiency, suggesting potential utility of lysosome-based therapies for *GRN*-associated diseases.

**Supplementary Information:**

The online version contains supplementary material available at 10.1038/s41598-025-12268-0.

## Introduction

Progranulin is a secreted pro-protein that is expressed by many cell types throughout the body^1–3^. Progranulin is critical for maintaining brain health, as loss-of-function mutations in progranulin (*GRN*) cause dominantly-inherited frontotemporal dementia (FTD), a neurodegenerative disorder comprising several clinical syndromes that affect behavior and language^4,5^. Other *GRN* variants are associated with increased risk for FTD^6^, Alzheimer’s disease, ^7^and Parkinson’s disease^8^. Progranulin is trafficked to lysosomes^9,10^ and is necessary for maintaining lysosomal function, as people with loss-of-function mutations on both *GRN* alleles, resulting in nearly complete progranulin deficiency, develop the lysosomal storage disorder Neuronal Ceroid Lipofuscinosis (NCL)^11–14^.

Progranulin maintains lysosomal function through several mechanisms, which may be mediated both by the intact protein and by the seven distinct granulin fragments generated by proteolytic cleavage of progranulin^15^. Progranulin and some granulins interact with the protease cathepsin D and enhance its maturation, stability, and activity^16–20^. Progranulin also regulates several enzymes and co-factors involved in sphingolipid metabolism. Progranulin interacts with β-hexosaminidase^21^ and β-glucocerebrosidase^22–25^ and promotes their activity. For β-glucocerebrosidase, progranulin recruits chaperones to the enzyme and facilitates proper folding and glycosylation^22,23^. Progranulin also interacts with prosaposin, a pro-protein that is cleaved into necessary co-factors for sphingolipid metabolism^26^and facilitates its trafficking to lysosomes^10,27^. Finally, progranulin appears to be necessary for maintaining lysosomal levels of bis(monoacylglycero)phosphate (BMP), a lipid that is a critical co-factor for sphingolipid metabolism^26,28^.

Consistent with these functions, people with *GRN* mutations and progranulin-insufficient mouse models exhibit signs of impaired lysosomal proteolysis and sphingolipid metabolism. People with mutations on one^29,30^ or both^11–14^ *GRN* alleles accumulate more lipofuscin than age-matched controls. Similarly, *Grn*^*–/–*^ mice accumulate elevated levels of autofluorescent lipofuscin^31,32^ and immunolabeling for SCMAS (subunit C of mitochondrial ATP synthase)^33^a marker of storage material in NCL^34–36^. Homozygous *Grn*^*R493X/R493X*^ mice, which model a pathogenic *GRN* mutation, are also progranulin-deficient and accumulate lipofuscin^37^. People with *GRN* mutations, as well as *Grn*^*–/–*^ or *Grn*^*R493X/R493X*^ mice, accumulate sphingolipids such as gangliosides^38^ and glucosylsphingosine^28^.

In addition to storage material, brain tissue from people with *GRN* mutations contains increased levels of lysosomal membrane proteins and enzymes such as cathepsin D^39^. These increases are most prominent in degenerated brain regions, with spared brain regions exhibiting mild lysosomal changes^30^. Brains of progranulin-insufficient mice also exhibit increases in lysosomal proteins^33,37,39–41^and increases in the number of lysosomes in neurons^42^ and microglia^43,44^. In patients and mouse models, these changes are associated with increased expression of lysosomal transcripts^30,41–45^. This might be mediated by TFEB or related transcription factors such as TFE3 or MiTF, which activate expression of lysosomal transcripts under conditions of lysosomal stress^46–48^. Consistent with this possibility, progranulin knockdown increases nuclear TFEB localization in neuroblastoma cells^49^, and microglia from *Grn*^*–/–*^ mice exhibit increased levels of nuclear TFEB and TFE3^43,50^. Though this response may be an attempt to compensate for lysosomal impairment^51–54 ^, overproduction of lysosomal proteases may contribute to development of FTD-related pathology in *Grn*^*–/–*^ mice^40^. Thus, brains of people with *GRN* mutations exhibit complex lysosomal dysregulation characterized by accumulation of storage material and lysosomal proteins, coupled with potentially detrimental excess activity of some proteases.

To investigate the role of lysosomal biogenesis in FTD-*GRN* and NCL-*GRN*, we investigated TFEB’s effects in progranulin-deficient cells and mice. We hypothesized that TFEB’s activation of lysosomal biogenesis^46,47^ and/or promotion of lysosomal exocytosis^51^ might have protective effects in these model systems. However, it could also be possible that producing additional progranulin-insufficient lysosomes would have detrimental effects in these models. We therefore transfected *GRN* knockout HEK-293 cells with a TFEB-GFP plasmid^55^ and transduced the thalamus of *Grn*^*–/–*^ mice with an AAV-TFEB vector to determine the effects of TFEB overexpression in progranulin-insufficient models.

## Results

### GRN knockout HEK-293 cells have abnormal autophagic flux

**Fig. 1:**
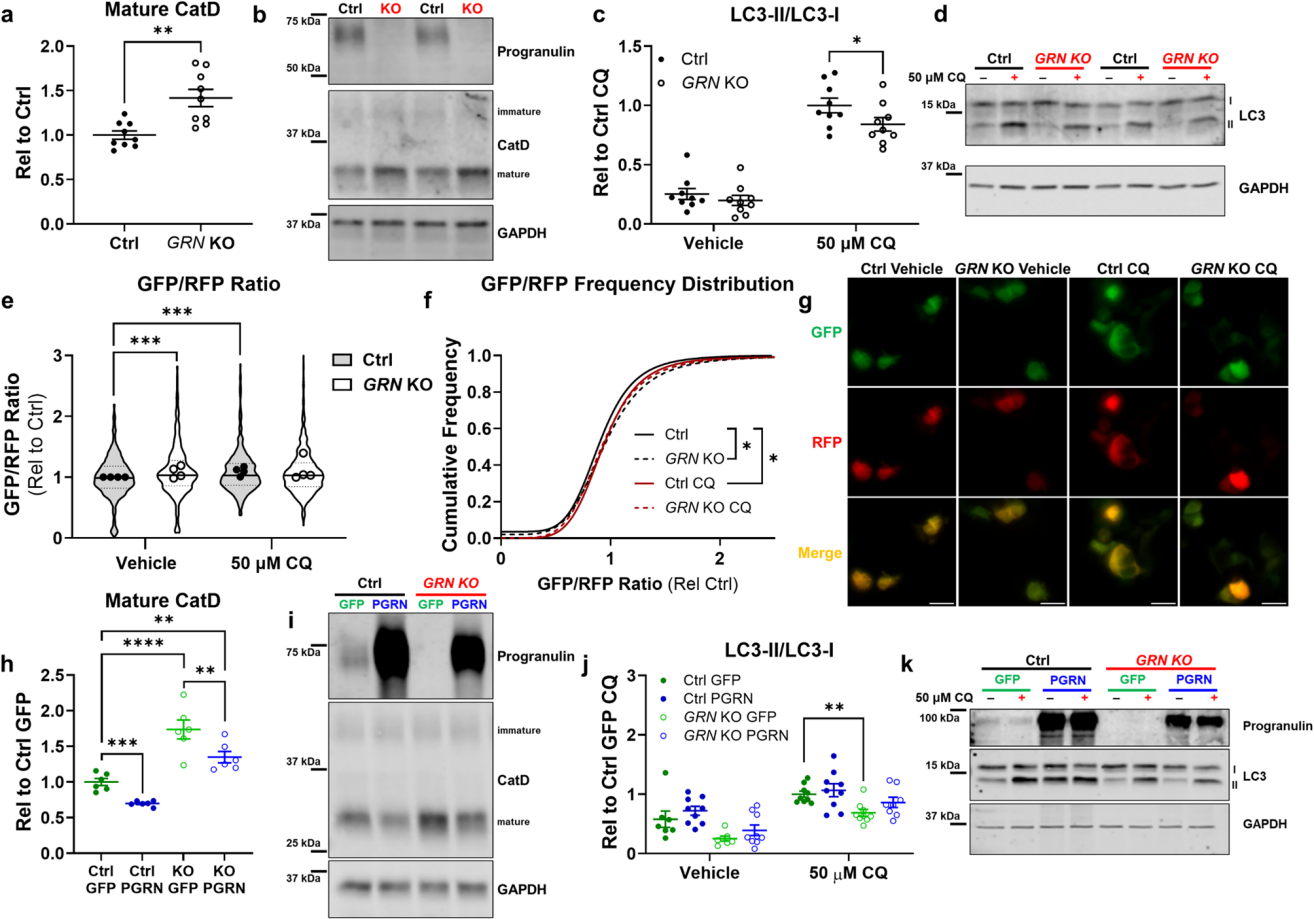
*GRN* Knockout HEK-293 Cells Exhibit Autophagy-Lysosomal Abnormalities. We deleted the entire *GRN* coding sequence from HEK-293 cells using CRISPR-Cas9, resulting in complete loss of progranulin. (**a**,** b**), Lysates of *GRN* knockout (*GRN* KO) cells had elevated levels of mature cathepsin D (CatD, *t* test, *p* = 0.0013, *n* = 9 per group). (**c**,** d**), *GRN* KO cells also exhibited a deficit in autophagy, defined as a reduced LC3-II/LC3-I ratio after incubating for one hour with 50 µM chloroquine (CQ) (ANOVA effect of CQ, *p* < 0.0001, * = *p* = 0.0406 by Holm-Sidak post-hoc test, *n* = 9 per group). (**e**–**g**), Fluorescent imaging of cells transfected with GFP-LC3-RFP-LC3ΔG confirmed a reduction in autophagic flux in *GRN* KO cells. The ratio of GFP/RFP fluorescence was significantly increased in *GRN* KO cells versus controls (**e**, linear mixed effects model effect of *GRN*, *p* = 0.0002, *** = *p* < 0.001 by Tukey’s post-hoc test, *n* = 528–594 cells per group from 4 independent cultures). Additionally, incubation with 50 µM chloroquine for 3 h increased the GFP/RFP ratio in control cells (**e**, linear mixed effects model effect of CQ, *p* < 0.0001, *** = *p* < 0.001 by Tukey’s post-hoc test) but not in *GRN* KO cells (*p* = 0.534 by Tukey’s post-hoc test). Data in (**e**) are shown as the median fluorescent intensity normalized to control vehicle-treated cells from each culture, with violin plots showing the distribution of all cells measured and symbols showing the median value for each culture^108^. (**f**), Similar results were obtained by analyzing the frequency distribution of the GFP/RFP ratio in cells transfected with GFP-LC3-RFP-LC3ΔG (**f**, Kolmogorov-Smirnov test, control vehicle vs. *GRN* KO vehicle, *p* = 0.0147, control vehicle vs. control CQ, *p* = 0.0472, *GRN* KO vehicle vs. *GRN* KO CQ, *p* = 0.7649). Scale bars in (**g**) represent 20 μm. (**h**,** i**), Restoration of progranulin (PGRN) to *GRN* KO cells reduced levels of mature CatD, though CatD still remained elevated compared to control cells (ANOVA effect of *GRN* KO, *p* < 0.0001, effect of PGRN expression, *p* < 0.0001, ** = *p* < 0.01, *** = *p* < 0.001, **** = *p* < 0.0001 by Holm-Sidak post-hoc test, *n* = 6 per group). (**g**, **h**), Restoration of PGRN to *GRN* KO cells also normalized the LC3-II/LC3-I ratio (ANOVA effect of PGRN expression, *p* = 0.0328, *n* = 7–9 per group), as GFP-transfected *GRN* KO cells had lower LC3-II/LC-I after chloroquine treatment than control cells (** = *p* = 0.0092 by Holm-Sidak test) while PGRN-transfected *GRN* KO cells did not differ from controls (*p* = 0.3058 by Holm-Sidak test). Full images of immunoblots are shown in Fig. S2.

We generated *GRN* knockout (*GRN* KO) HEK-293 cells using CRISPR-Cas9, with guide RNAs flanking the *GRN* coding sequence^56^. After screening clones with PCR to confirm deletion of the *GRN* coding sequence, we isolated a line exhibiting complete loss of progranulin (Fig. 1b). A control line was generated in parallel to *GRN* KO cells using guide RNA targeting *LacZ*. *GRN* KO HEK-293 cells exhibited lysosomal abnormalities such as an increase in levels of mature cathepsin D (Fig. 1a, b), which has also been reported in brain tissue from patients with FTD-*GRN*^30,39^ and *Grn*^*–/–*^ mice^33,39^. *GRN* KO cells also exhibited signs of impaired autophagy, with a lower LC3-II/LC3-I ratio than control cells after incubating for one hour with 50 µM chloroquine (Fig. 1c, d). This phenotype is similar to the impaired autophagy reported in *Grn*^*–/–*^ primary macrophages and neurons^57^.

To further investigate autophagy, we transfected control and *GRN* KO cells with GFP-LC3-RFP-LC3ΔG (Addgene #168997^58^). This construct allows for assessment of autophagic flux, as GFP-LC3-I is subject to lipidation and degradation through the autophagy-lysosomal pathway, while RFP-LC3ΔG cannot be lipidated and degraded^58^. Thus, the GFP/RFP fluorescence ratio increases under conditions of lower autophagic flux. We found that *GRN* KO cells had a higher GFP/RFP ratio than control cells, and that incubation with 50 µM chloroquine for three hours increased the GFP/RFP ratio in control cells but not in *GRN* KO cells (Fig. 1e–g). These results indicate lower autophagic flux in *GRN* KO cells. We also analyzed immunoreactivity of endogenous LC3 in control and *GRN* KO cells, and found that *GRN* KO cells had fewer LC3-immunoreactive puncta than control cells (Fig. S1), with a generally more diffuse pattern of LC3 immunostaining. Collectively, these data suggest lower autophagic flux in *GRN* KO cells, with potential impairment in both autophagosome formation (lower LC3 immunoreactivity and LC3-II/LC3-I by immunoblot) and impaired degradation of autophagosomes (GFP-LC3-RFP-LC3ΔG imaging).

To confirm that these phenotypes were due to loss of progranulin, we restored progranulin to *GRN* KO cells using a plasmid expressing human progranulin (PGRN). Restoration of PGRN partially normalized both cathepsin D levels (Fig. 1h, i) and the LC3-II/LC3-I ratio (Fig. 1j, k) in *GRN* KO cells, showing that these are progranulin-dependent phenotypes. Based on these data, we concluded that *GRN* KO HEK-293 cells model aspects of autophagy-lysosomal dysfunction observed in patients with FTD-*GRN* and progranulin-insufficient experimental models.

### TFEB overexpression normalizes autophagic flux in GRN KO cells

**Fig. 2:**
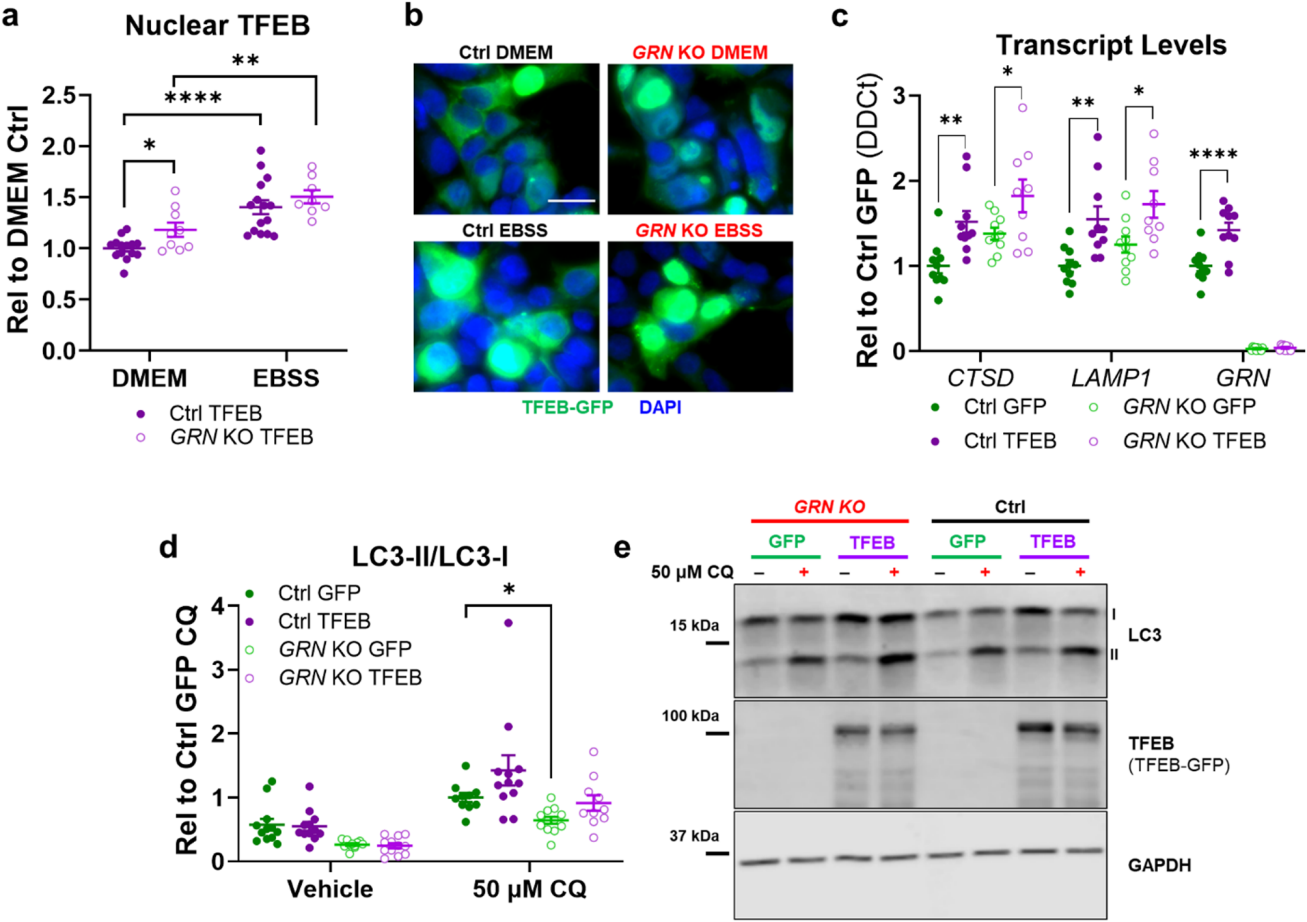
TFEB Overexpression Increases Expression of Lysosomal Transcripts and Normalizes Autophagy in *GRN* Knockout HEK-293 Cells. (**a**,** b**), When transfected with a TFEB-GFP construct, a higher proportion of *GRN* KO cells than controls exhibited nuclear TFEB localization under standard culture conditions (ANOVA effect of *GRN*, *p* = 0.0133, * = *p* = 0.0357 by Holm-Sidak test, *n* = 9–11/group). However, both control and *GRN* KO cells exhibited an increase in nuclear TFEB localization after nutrient starvation by incubating for one hour in EBSS (ANOVA effect of EBSS, *p* < 0.0001, ** = *p* = 0.005 and **** = *p* < 0.0001 by Holm-Sidak test). (**c**), *GRN* KO cells exhibited higher expression of lysosomal transcripts such as *CTSD* than controls, with a similar trend for *LAMP1* (MANOVA effect of *GRN*, *p* < 0.001, *CTSD* ANOVA effect of *GRN*, *p* = 0.0087, *LAMP1* ANOVA effect of *GRN*, *p =* 0.0895). Transfection with TFEB-GFP increased lysosomal transcripts in both control and *GRN* KO cells versus transfection with a GFP control plasmid (MANOVA effect of TFEB, *p* < 0.001, * = *p* < 0.05, ** = *p* < 0.01, **** = *p* < 0.0001 by Holm-Sidak post-hoc test, *n* = 9–10 samples/group). (**d**,** e**), TFEB overexpression also normalized the autophagy deficits of *GRN* KO cells (3-way ANOVA effect of CQ, *p* < 0.0001, effect of *GRN*, *p* < 0.0001, CQ x TFEB, *p* < 0.0282, *n* = 10–12/group), as GFP-transfected *GRN* KO cells had lower LC3-II/LC-I after chloroquine treatment than control cells (* = *p* = 0.0266 by Holm-Sidak test), while TFEB-transfected *GRN* KO cells did not (*p* = 0.3923 by Holm-Sidak test). The scale bar in **b** represents 20 μm. Full images of immunoblots are shown in Fig. S3.

The elevated cathepsin D of *GRN* KO cells mimics that of progranulin-deficient mice^33,39^, which exhibit increased expression of *Ctsd* transcript that may be driven by TFEB-family transcription factors^30,41–44,50^. To investigate whether *GRN* KO cells also exhibit signs of increased TFEB activity, we transfected *GRN* KO and control cells with a TFEB-GFP plasmid (Addgene #38119^55^). Consistent with reports from other cell types^43,49,50^, a higher percentage of *GRN* KO cells than control cells exhibited nuclear TFEB localization (Fig. 2a, b). Nutrient starvation increased nuclear TFEB localization in both control and *GRN* KO cells (Fig. 2a, b), showing that despite the increased baseline levels of nuclear TFEB, *GRN* KO cells are able to further increase nuclear TFEB localization in response to appropriate stimuli.

We next investigated whether enhancing TFEB activity would normalize the autophagic deficits of *GRN* KO cells. We transfected cells with TFEB-GFP or a similar GFP plasmid (Takara #632469), and found that TFEB-GFP increased expression of *CTSD* and *LAMP1* in both control and *GRN* KO cells (Fig. 2c), showing that TFEB overexpression enhanced transcription of TFEB target genes. We next analyzed the effects of TFEB-GFP on autophagy. GFP-transfected *GRN* KO cells exhibited a lower LC3-II/LC3-I ratio than controls after chloroquine treatment, while the LC3-II/LC3-I ratio of *GRN* KO cells transfected with TFEB-GFP did not differ from controls (Fig. 2d, e). These data indicate that enhancing lysosomal biogenesis in *GRN* KO cells can improve autophagy deficits, despite the fact that their lysosomes remain progranulin-deficient.

### AAV-TFEB increases lysosomal protein levels in the brain

**Fig. 3:**
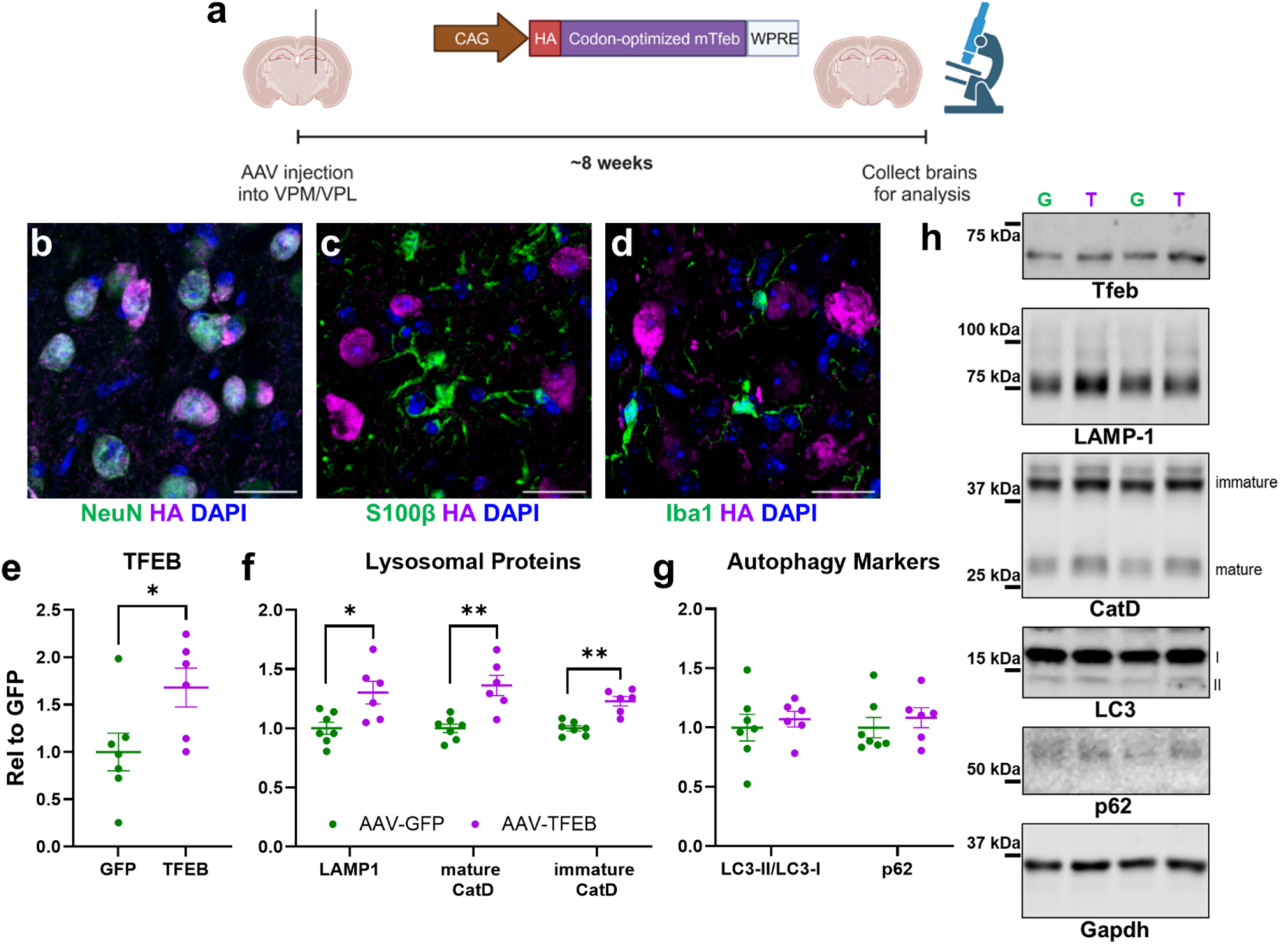
TFEB Overexpression in Wild-type Mouse Brain Increases Levels of Lysosomal Proteins. (**a**), We injected AAV2 vectors expressing either GFP or HA-tagged codon-optimized mouse TFEB into the ventroposteromedial/lateral nuclei of the thalamus (VPM/VPL) of wild-type mice. After 8 weeks, we collected brains for analysis of TFEB expression and lysosomal proteins. (**b–d**), AAV-TFEB primarily transduced neurons, based on immunostaining for the HA tag and markers of neurons (NeuN), astrocytes (S100β), and microglia (Iba1). (**e**), TFEB overexpression in thalamus was further confirmed by immunoblot (*t* test, *p* = 0.0373). (**f**), Immunoblot of thalamic tissue confirmed increases in lysosomal proteins in TFEB-treated mice (MANOVA effect of TFEB, *p* = 0.006), with increases in LAMP-1 (*t* test, *p* = 0.0141), mature cathepsin D (*t* test, *p* = 0.0017), and immature cathepsin D (*t* test, *p* = 0.0003). (**g**), However, analysis of the LC3-II/LC3-I ratio (*t* test, *p* = 0.6163) and p62 (*t* test, *p* = 0.5056) did not reveal signs of increased autophagy. *n* = 6–7 mice per group. Scale bars in (**b–d**) represent 25 μm. Panel (**a**) created using Biorender.com. (Arrant, A. (2025) https://BioRender.com/flnev93 and https://BioRender.com/6cia20y). Full images of immunoblots are shown in Fig. S4.

To investigate the effects of TFEB overexpression in vivo, we developed an AAV2 vector expressing codon-optimized mouse TFEB (Fig. 3a). When injected into the ventral posteromedial/lateral thalamus (VPM/VPL) of wild-type C57Bl/6J mice, AAV-TFEB transduced mostly neurons (Fig. 3b–d). Many transduced neurons exhibited nuclear TFEB localization (Fig. 3b), based on co-localization with the neuronal marker NeuN and the nuclear marker DAPI. When analyzed approximately 8 weeks after AAV injection, thalamic lysates from AAV-TFEB–treated mice exhibited increased TFEB levels compared to mice treated with AAV-GFP, as well as increases in LAMP-1 and cathepsin D (Fig. 3e, f). However, AAV-TFEB did not significantly alter levels of the autophagy markers LC3-II or p62 in these thalamic lysates (Fig. 3g). These data indicate that injecting AAV-TFEB into mouse thalamus increases lysosomal biogenesis, likely in neurons.

### AAV-TFEB reduces lysosomal storage material in Grn^–/–^ thalamus

We next investigated the effects of AAV-TFEB on lysosomal storage phenotypes of *Grn*^*–/–*^ mice by injecting AAV-TFEB or AAV-GFP into the ventroposteromedial/lateral thalamus (VPM/VPL), which develops progressively worsening lysosomal abnormalities and inflammation as *Grn*^*–/–*^ mice age^31,32,44,59,60^. Only *Grn*^*–/–*^ mice were included in this study, as the accumulation of lysosomal storage material and increase in inflammatory markers have been repeatedly documented at the ages used in this study^61^. *Grn*^*–/–*^ mice were injected at 6–7 months of age, at which point they are in the early stages of lysosomal storage pathology^31^, and analyzed approximately 8 weeks later. In a prior study using AAV-*Grn* to restore progranulin to *Grn*^*–/–*^ mice, we found that 8 weeks was sufficient to reduce lysosomal storage pathology and inflammation^33^. Other *Grn*^*–/–*^ littermates were administered AAV-GFP or left uninjected to serve as control groups.

**Fig. 4:**
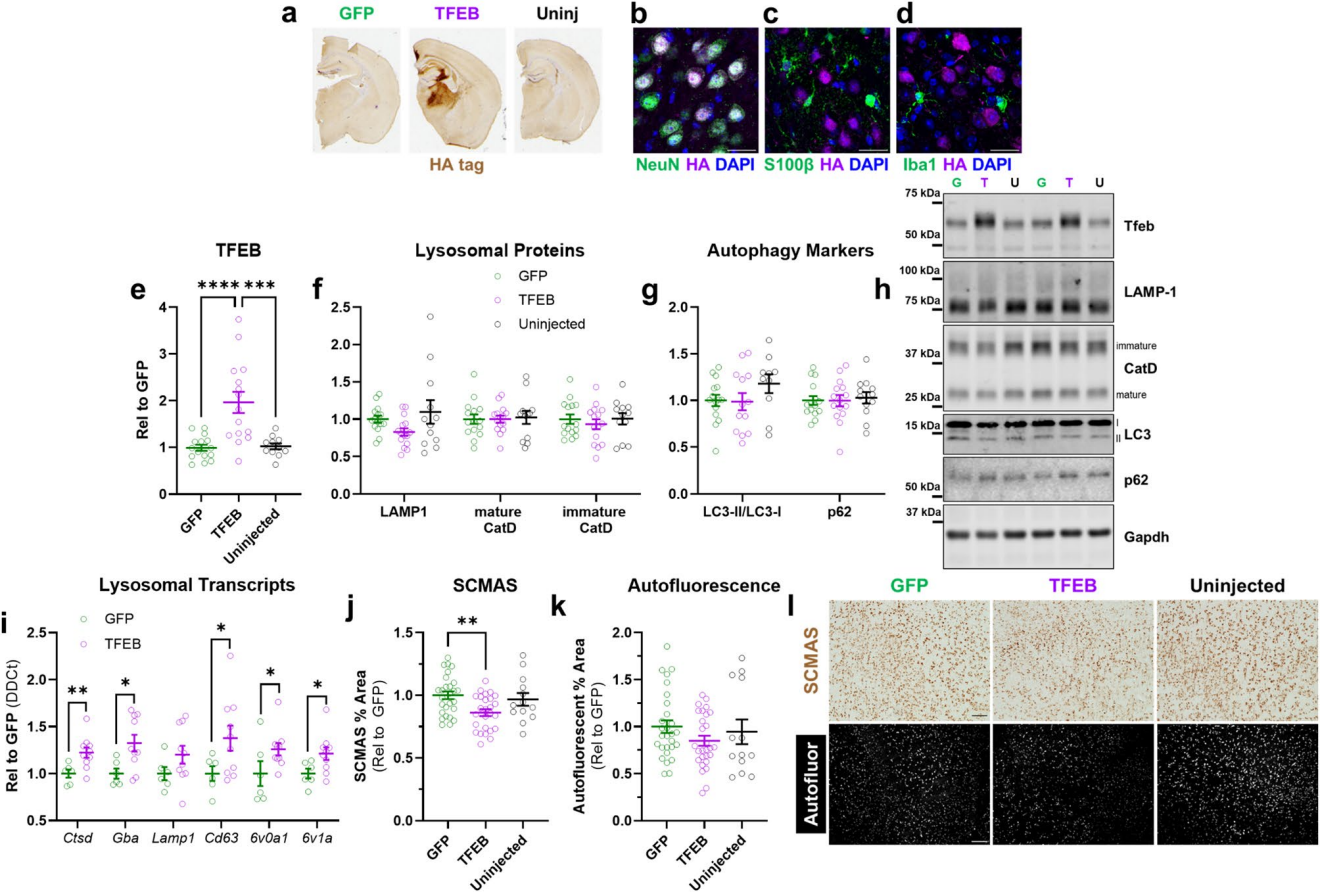
TFEB Overexpression Reduces Lysosomal Storage Material in *Grn*^*–/–*^ Mice. (**a**), AAV-TFEB expressed HA-tagged TFEB throughout the ventroposteromedial/lateral thalamic nuclei of *Grn*^*–/–*^ mice. (**b**–**d**), Similar to wild-type mice, AAV-TFEB primarily transduced neurons and increased TFEB levels in the thalamus (**e**, ANOVA effect of TFEB, *p* < 0.0001, *** = *p* = 0.0001 and **** = *p* < 0.0001 by Holm-Sidak post-hoc test). (**f**), In contrast to wild-type mice, AAV-TFEB did not increase LAMP-1 or cathepsin D (CatD) in *Grn*^*–/–*^ mice (MANOVA effect of TFEB, *p* = 0.467). AAV-TFEB also did not alter the LC3-II/LC3-I ratio (**g**, ANOVA effect of TFEB, *p* = 0.2413) or p62 levels (**g**, ANOVA effect of TFEB, *p* = 0.9191) in *Grn*^*–/–*^ mice. For (**e**–**g**), *n* = 10–15 mice/group. **i**, However, AAV-TFEB did increase levels of multiple lysosomal transcripts as measured by qPCR (MANOVA effect of TFEB, *p* = 0.04, * = *p* < 0.05, ** = *p* < 0.01 by *t* test, *n* = 6–10 samples/group). (**j**), AAV-TFEB reduced immunoreactivity for SCMAS, a marker of lysosomal storage material that is elevated in *Grn*^*–/–*^ thalamus (ANOVA effect of TFEB, *p* = 0.0063, * = *p* = 0.0039 by Holm-Sidak post-hoc test, *n* = 13–27 mice/group). (**k**), AAV-TFEB produced a similar trend for autofluorescent lipofuscin (ANOVA effect of TFEB, *p* = 0.2730), which exhibited greater variability, especially in the uninjected group. Representative 10X images of SCMAS immunostaining and autofluorescence are shown in **l** with 100 μm scale bars. SCMAS = subunit C of mitochondrial ATP synthase. Full images of immunoblots are shown in Fig. S5.

AAV-TFEB expressed TFEB throughout the VPM/VPL (Fig. 4a). As in wild-type mice, AAV-TFEB mostly transduced neurons in *Grn*^*–/–*^ mice (Fig. 4b–d) and increased TFEB levels in thalamic lysates (Fig. 4e). However, AAV-TFEB did not increase LAMP-1 or cathepsin D protein levels in *Grn*^*–/–*^ mice (Fig. 4f, h), nor did it alter levels of LC3-II or p62 (Fig. 4g, h). To determine if this lack of effect on autophagy-lysosomal protein levels indicated a lack of TFEB transcriptional activity, we analyzed levels of several transcripts that are regulated by TFEB^62^. AAV-TFEB increased expression of nearly all of these transcripts relative to AAV-GFP (Fig. 4i), suggesting that TFEB is transcriptionally active in *Grn*^*–/–*^ mice. The lack of increase in LAMP-1 and cathepsin D protein might therefore be related to the fact that both of these proteins are already elevated in *Grn*^*–/–*^ mice relative to wild-type^33,39^. Alleviating lysosomal storage phenotypes might thus reduce levels of these proteins through either clearance of lysosomal storage material or by reducing activation of endogenous TFEB or related transcription factors. Either of these effects could counteract the increased synthesis of these proteins due to increased AAV-TFEB–mediated transcription.

To determine if AAV-TFEB reduced lysosomal storage material in *Grn*^*–/–*^ mice, we analyzed levels of the lipofuscin marker SCMAS^34–36^ and levels of autofluorescent lipofuscin, both of which are elevated in *Grn*^*–/–*^ thalamus^31–33,44,59^. AAV-TFEB significantly reduced levels of SCMAS versus AAV-GFP (Fig. 4j, l), with a similar trend for reduction of SCMAS versus uninjected mice (Holm-Sidak post-hoc test, *p* = 0.05). AAV-TFEB also produced a trend for reduction of autofluorescent lipofuscin (Fig. 4k, l), which was more variable in all groups. These data show that TFEB overexpression activates lysosomal biogenesis and reduces levels of lysosomal storage material in the thalamus of *Grn*^*–/–*^ mice.

### Gliosis at the AAV injection site

We next attempted to determine if the improvement in lysosomal storage pathology with AAV-TFEB treatment in *Grn*^*–/–*^ mice would improve other aspects of *Grn*^*–/–*^ pathology. We immunostained for CD68 and Galectin 3, markers of reactive microglia/macrophages in *Grn*^*–/–*^ mice^59,63^. We found that AAV-GFP increased CD68 immunoreactivity in the thalamus relative to uninjected *Grn*^*–/–*^ mice, and produced a similar trend in Galectin 3 immunoreactivity (Fig. S6). AAV-TFEB induced less CD68 immunoreactivity than AAV-GFP, but the pattern of staining also indicated an inflammatory response to AAV injection. Thus, due to the apparent reaction of *Grn*^*–/–*^ mice to some aspect of AAV injection, we were unable to determine whether TFEB overexpression could improve gliosis in *Grn*^*–/–*^ mice.

## Discussion

These data show that TFEB overexpression is capable of improving autophagy-lysosomal deficits in progranulin-insufficient models. In *GRN* KO HEK-293 cells, TFEB overexpression increased lysosomal transcript levels and stimulated autophagy. In the thalamus of *Grn*^*–/–*^ mice, TFEB overexpression increased lysosomal transcripts and reduced levels of SCMAS, a marker of lysosomal storage material. Progranulin insufficiency induces an increase in nuclear localization of TFEB and related transcription factors, as we observed in *GRN* KO cells (Fig. 2b) and others have reported in *Grn*^*–/–*^ mice^43,49,50^. This may drive the increases in lysosomal transcripts^30^ and proteins^39^ observed in patients with *GRN* mutations and in *Grn*^*–/–*^ mice. While this transcriptional response does not prevent accumulation of storage material and sphingolipids^11,28–30,38, ^, this study indicates that further enhancing activity of TFEB can improve autophagy-lysosomal deficits caused by progranulin insufficiency.

The protective effects of TFEB in *GRN* KO cells and *Grn*^*–/–*^ mice are consistent with many studies showing beneficial effects of TFEB overexpression in models of lysosomal storage disorders and neurodegenerative diseases. TFEB overexpression reduces levels of storage material and improves functional outcomes in models of lysosomal storage diseases such as Pompe disease, multiple sulfatase deficiency, and mucopolysaccharidosis^51–53^. TFEB overexpression also promotes clearance of lipofuscin in fibroblasts from patients with Batten’s disease, a form of NCL^51^. In mouse models of neurodegenerative disorders, TFEB overexpression reduces the accumulation of amyloid β^64–66, ^tau^67,68^, and α-synuclein^69,70^ pathology.

The protective effects of TFEB may be mediated by several mechanisms. TFEB’s activation of lysosomal biogenesis and stimulation of autophagy may mediate its protective effects against neurodegenerative pathology^71,72^. The normalization of autophagy in *GRN* KO cells transfected with TFEB-GFP may be due to TFEB’s stimulatory effects on autophagy and lysosomal biogenesis^47^. Whether TFEB’s stimulation of autophagy-lysosomal activity is responsible for reducing storage material in *Grn*^*–/–*^ thalamus is less clear. AAV-TFEB elevated lysosomal transcript levels in *Grn*^*–/–*^ thalamus, consistent with an increase in lysosomal biogenesis. However, the lack of increase in lysosomal proteins or change in autophagy markers makes it unclear if the increased transcription resulted in greater lysosomal activity or autophagic flux. This may be due to balancing the increased synthesis of new lysosomal proteins with clearance of storage material, which in other studies has been associated with a reduction in lysosomal size^52^.

Lysosomal exocytosis may be another mechanism by which TFEB overexpression reduced storage material in *Grn*^*–/–*^ mice, as TFEB-mediated clearance of storage material in models of other lysosomal storage disorders has been associated with increased lysosomal exocytosis^51,52^. Lysosomal exocytosis may already be increased in *Grn*^*–/–*^ mice and patients with FTD-*GRN*, as both exhibit increases in extracellular vesicles in brain tissue^73^. This might allow neurons or glia from *Grn*^*–/–*^ mice to clear storage material without needing to degrade it through the autophagy-lysosomal pathway.

An important caveat to the reduction in lysosomal storage material in AAV-TFEB–treated *Grn*^*–/–*^ mice is the modest effect size. We observed a 14–15% reduction of SCMAS and autofluorescence compared to the roughly 50% reduction of autofluorescence we observed in a prior study with AAV-*Grn* under similar conditions^33^. However, it is notable that we administered AAV-TFEB to *Grn*^*–/–*^ mice at an age when they already carry a significant burden of storage pathology^31,32,59^. It is possible that earlier treatment with AAV-TFEB or analysis after a longer period of TFEB overexpression would result in a greater reduction in storage material than observed in this study.

A technical problem that limited the available outcome measures in the current study was the AAV-related inflammation that occurred in *Grn*^*–/–*^ mice (Fig. S6). *Grn*^*–/–*^ mice have a well-documented hyper-inflammatory phenotype^74,75^, and we and others have encountered immune responses to AAV injection in *Grn*^*–/–*^ mice^33,76^. Future studies using less immunogenic techniques may provide a way to circumvent this issue in *Grn*^*–/–*^ mice^77,78^.

An interesting area for future investigation will be the role of TFEB and related transcription factors in other forms of FTD. In addition to *GRN*, FTD-causing mutations occur in several other genes involved in aspects of autophagy-lysosomal or endolysosomal function, including *CHMP2B*^79, ^*VCP*^80, ^*TBK1*^81–83^, *OPTN*^83, ^and *C9ORF72*^84,85^. Patients with *CHMP2B* mutations accumulate lysosomal storage material^86^, and experimental models of several genetic FTD subtypes exhibit accumulation of lysosomal proteins^86–88^. Degenerated brain regions of patients with sporadic forms of FTD also accumulate lysosomal proteins and storage material^30,89^. Thus, dysregulation of autophagy-lysosomal function may be a feature of multiple FTD subtypes^90,91^.

In conclusion, this study indicates that increasing TFEB activity alleviates the autophagy deficits and accumulation of lysosomal storage material caused by progranulin insufficiency. This suggests that the increase in nuclear TFEB localization and increased levels of lysosomal proteins observed in *Grn*^*–/–*^ mice^43,49,50^ and patients with FTD-*GRN*^39^ may be a protective response to loss of progranulin’s effects in lysosomes. However, these changes may also contribute to FTD-related pathology. For example, overproduction of the lysosomal protease legumain may contribute to TDP-43 dysregulation in *Grn*^*–/–*^ mice^40^. It is also unclear if exocytosis of endolysosomal cargo contributes to inflammation in FTD-*GRN*, which may be a key mechanism of disease^44,60,92–94^. Endolysosomal exocytosis and the resulting extracellular vesicles may also contribute to spread of pathology, including TDP-43^95,96^. It will therefore be important for future studies to further define the role of TFEB and related transcription factors in pathogenesis of FTD-*GRN* and other forms of FTD.

## Methods

### Cell culture

HEK-293 cells (#CRL-1573, ATCC) and the control and *GRN* KO cells derived from these cells, were cultured in DMEM (Corning Life Sciences) with 10% fetal bovine serum (Premium Select, R&D Systems), and 1% penicillin/streptomycin (ThermoFisher) at 37ºC and 5% CO_2_. Cells were cultured at uniform density in 24-well plates (Corning) for all experiments. For experiments involving cellular imaging, cells were plated onto No. 1.5 circular glass coverslips (Electron Microscopy Sciences) that were pre-coated with poly-D-lysine (0.1 mg/mL). For all procedures described below, cells were transfected using Fugene HD transfection reagent (Promega) using the manufacturer’s instructions.

### Generation of GRN knockout HEK-293 cells (GRN KO cells)

We employed a CRISPR-Cas9 strategy using two guide RNAs to delete the entire *GRN* coding sequence from HEK-293 cells^56^. Guide RNAs targeted to sites flanking the *GRN* coding sequence were designed at crispr.mit.edu^97^ (sgRNA1 = GCAAAGTACCAAGGAACGTC, sgRNA2 = CCCTCGGGACCCCACTCGGA). These sgRNAs were cloned into the pX330-U6-Chimeric_BB-CBh-hSpCas9 vector (Addgene #42230, a gift from Feng Zhang)^98^. HEK-293 cells were co-transected with equivalent amounts of each *GRN* sgRNA vector and a GFP plasmid (Takara #632469) to determine transfection efficiency. Control cells were transfected with the same Cas9 vector containing an sgRNA targeting *LacZ* (TGCGAATACGCCGGGGCGAT)^99^ and the same GFP plasmid. The cells with brightest GFP fluorescence were isolated by FACS, then subjected to limiting dilution and clonal expansion. Cells were genotyped for *GRN* deletion using the following primers: F – AGACTCCACTGGCCACCATA, WT R – CTCCTCTGGCCAATCCAAGAT, KO R – GAGGGGATGGCAGCTTGTAA.

### GFP-LC3-RFP-LC3ΔG transfection and imaging

Control and *GRN* KO cells were transfected with a plasmid expressing GFP-LC3-RFP-LC3ΔG (pcDNA3-GFP-LC3-RFP-LC3ΔG, Addgene # 168997, a gift from Noboru Mizushima^58^). Two days after transfection, cells were changed to media with or without 50 µM chloroquine and incubated at 37º C for three hours before fixing with 4% paraformaldehyde/4% sucrose in PBS. Coverslips were mounted onto slides with Prolong Gold reagent with DAPI (ThermoFisher), then imaged on an EVOS M5000 microscope (ThermoFisher). Four 20X z-stacks were taken from different areas of each coverslip.

The ratio of GFP/RFP fluorescence was analyzed using Cell Profiler^100^. Cellular ROIs were defined using red fluorescence, as the GFP-LC3-RFP-LC3ΔG plasmid generates RFP as an internal control^58^. These ROIs were then overlaid onto the green fluorescent channel and used to calculate median fluorescence on a cell-by-cell basis. All imaging and analysis were done by an investigator blinded to genotype and treatment of the cultures.

### TFEB-GFP transfection and imaging

Control and *GRN* KO cells were transfected with a plasmid expressing TFEB-GFP (pEGFP-N1-TFEB, Addgene #38119, a gift from Shawn Ferguson^55^). Three days after transfection, cells were subjected to a full media change to either fresh culture media or to EBSS (Cytiva), incubated at 37 ºC for one hour, then fixed with 4% paraformaldehyde/4% sucrose in PBS. Coverslips were mounted onto slides with Prolong Gold reagent with DAPI (ThermoFisher), then imaged on an EVOS M5000 microscope (ThermoFisher). Four 20X images were taken from different areas of each coverslip, and the number of GFP + cells with and without nuclear GFP (defined by colocalization with DAPI) was manually counted using ImageJ.

### LC3 Immunostaining and imaging

Control and *GRN* KO cells were plated onto coverslips and cultured for approximately 24 h before fixing with 4% paraformaldehyde/4% sucrose in PBS. Fixed cells were blocked in 3% BSA (Fisher Scientific) and 0.5% saponin (MilliporeSigma) in PBS, then incubated overnight with an LC3A/B antibody (Cell Signaling Technologies #12741) in the same solution. The following day, coverslips were washed in PBS before incubation with an AlexaFluor 594 donkey anti-rabbit antibody (Thermo Fisher) before mounting onto slides with Prolong Gold reagent.

Coverslips were imaged at 60X on a Nikon Ti2 C2 confocal microscope, with three z-stacks taken per coverslip. The total LC3-immunoreactive area and DAPI-positive area were calculated from each field of view using ImageJ^101^. The LC3 area was divided by the DAPI area to correct for cellular density, and the resulting values were averaged to give a single corrected LC3-immunoreactive area for each coverslip. All imaging and analysis were done by investigators blinded to the genotype of the cultures.

### Restoring progranulin to GRN KO cells

To confirm that phenotypes observed in *GRN* KO cells were caused by loss of progranulin, control and *GRN* KO HEK 293 cells were transfected with plasmids expressing GFP or human progranulin under the CAG promoter. The GFP plasmid was generated by replacing IRES-GFP in the CIGW AAV plasmid (CAG-IRES-GFP-WPRE-rBG)^102^ with GFP to generate a CAG-GFP-WPRE-rBG plasmid. The progranulin plasmid was similarly generated by replacing IRES-GFP in the CIGW vector with human *GRN* cDNA with a C-terminal HA tag. Cells were plated at uniform density onto 24-well plates (Corning Life Sciences), then transfected with either the GFP or progranulin plasmid the following day. Four days later, cells were analyzed for cathepsin D and LC3 as described below.

### Chloroquine treatment for LC3 analysis

Control and *GRN* KO cells were plated at uniform density onto 24-well culture plates (Corning Life Sciences) and analyzed for autophagic flux four days later. For analysis of the effects of progranulin or TFEB on autophagy, cells were transfected with the GFP or progranulin plasmids described above, or p-EGFP-N1-TFEB (Addgene #38119) or pAcGFP-N1 (Takara # 632469) and analyzed four days after transfection. To analyze autophagic flux, cells were incubated in culture media with 50 µM chloroquine (MP Biomedicals) or vehicle (1:1000 sterile water diluted in media) for one hour. The cells were then washed with PBS and lysed with Ripa buffer (50 mM Tris, 150 mM NaCl, 0.1% SDS, 1% Triton X-100, 0.5% sodium deoxycholate, 5 mM EDTA, pH 7.5). Lysates were sonicated at 50% power using a probe-tip sonicator (Fisher Scientific), then centrifuged at 21,100 x *g* at 4 °C. Supernatants were processed for immunoblot of LC3 as described below.

### Mice

C57Bl/6J (Jackson Laboratory #00664) or *Grn*^*–/–*^ mice (Jackson Laboratory #036771-JAX)^75^ bred onto a congenic C57Bl/6J background were used for mouse studies. *Grn*^*–/–*^ mice were generated through crosses of either *Grn*^*+/–*^ or *Grn*^*–/–*^ mice, and littermates were randomly allocated to the different AAV treatment groups. Male and female mice were used for all experiments. Mice were housed in a facility accredited by the Association for Assessment and Accreditation of Laboratory Animal Care International, with a 12-hour light/dark cycle and free access to food (Envigo #7917) and water. Mouse experiments were approved by the Institutional Animal Care and Use Committee of the University of Alabama at Birmingham, were performed in accordance with the AVMA guidelines on euthanasia, and are reported based on the ARRIVE guidelines^103^.

### AAV study design

Two studies were conducted using AAV-TFEB in mice. The goal of the first study was to validate TFEB expression and activity. This study was conducted with wild-type C57Bl6/J mice treated with either AAV-GFP or AAV-TFEB. The sample size was chosen based on prior experience with analyzing lysosomal protein levels in mouse brain tissue. The goal of the second study was to determine if AAV-TFEB could reduce lysosomal storage pathology in *Grn*^*–/–*^ mice. This study was conducted using only *Grn*^*–/–*^ mice, due to their highly reproducible accumulation of lysosomal storage material. *Grn*^*–/–*^ mice treated with AAV-GFP served as the primary control group, with uninjected mice providing an additional control for any non-specific effects of stereotaxic injection or AAV administration. *Grn*^*–/–*^ mice treated with AAV-TFEB were the experimental group for this study. This study included multiple cohorts of mice that were used to obtain several outcome measures. Brains were sliced into hemibrains for analysis, with all mice contributing one hemibrain for imaging of autofluorescence and immunostaining. This large sample size was chosen based on the high variability of autofluorescence in *Grn*^*–/–*^ brain tissue. The remaining hemibrain was used for analysis of either lysosomal proteins or transcripts. Sample sizes for these analyses were chosen based on prior experience with analyzing these measures in mouse brain tissue.

Male and female littermates were used for both studies. At weaning, mice were assigned unique identification numbers and placed into housing cages by sex. After aging to around six months, mice were assigned to treatment group in alternating order by identification number. This strategy allowed investigators to remain blind to treatment group and ensured that treatment groups were evenly distributed among housing cages. AAV injection was conducted in as short a time frame as possible (typically only a few days per cohort) on a cage-wise basis, resulting in a mixed order of injections. TFEB expression was confirmed in all AAV-TFEB treated mice to support inclusion in the study.

### Generation of AAV vectors

A DNA fragment comprising codon-optimized mouse TFEB cDNA with an N-terminal HA tag (Integrated DNA Technologies, Coralville, IA) was cloned into the CIGW AAV plasmid^102^ (CAG-IRES-GFP-WPRE-rBG) to generate CAG-HA-mTFEB-WPRE-rBG. The CAG-GFP-WPRE-rBG AAV plasmid described above was used as the AAV-GFP control plasmid. These plasmids were packaged into AAV2 vectors by Vectorbuilder, Inc (Chicago, IL).

### AAV injections and brain collection

AAV-TFEB or AAV-GFP were injected bilaterally into the ventroposteromedial/lateral (VPM/VPL) thalamus using stereotaxic surgery as previously described^33,104^. Stereotaxic coordinates were 1.3 mm posterior, ± 1.5 mm lateral from bregma, and − 3.5 mm from the dura. One µL of AAV at 5 × 10^11^ genomes/mL was injected into each hemisphere. Mice were allowed to recover for 8–9 weeks before being anesthetized with Pentobarbital (Fatal Plus, 200 mg/kg) and transcardially perfused with 0.9% saline. Brains were removed and bisected into hemibrains. The left hemibrain was fixed in 4% paraformaldehyde (MilliporeSigma) and the right hemibrain was frozen on dry ice for biochemical or qPCR analysis.

### Immunoblot

Cells were prepared for immunoblot as described above. For analysis of thalamic tissue, the VPM/VPL thalamus was dissected from 1 mm slices of frozen brain tissue. Tissue samples were then homogenized in lysis buffer (50 mM Tris, 150 mM NaCl, 1% Triton X-100, 0.1% sodium deoxycholate, 5 mM EDTA, pH 7.5) and centrifuged for 10 min at 5000 x *g* at 4 °C.

Lysates of cells or thalamic tissue were analyzed for total protein content by BCA assay (ThermoFisher), and uniform amounts of protein were processed for SDS-PAGE. Tissue lysates were diluted in 2X Laemmli buffer and heated at 95 °C for 5 min. Due to lower total protein concentration, cell lysates were diluted with 4X loading buffer (125 mM Tris, 8% lithium dodecyl sulfate, 40% glycerol, 0.16% Orange G) and heated for 15 min at 70 °C. All samples were subjected to SDS-PAGE on 10% or 15% Tris-glycine polyacrylamide gels (Bio-Rad). Proteins were then transferred to Immobilon-F PVDF membranes (MilliporeSigma), blocked with protein-free blocking buffer (ThermoFisher), and probed overnight with primary antibody.

Primary antibodies used in this study included: progranulin (MilliporeSigma # #HPA008763), cathepsin D (Santa Cruz #sc-6486), GAPDH (MilliporeSigma # MAB374), LC3 (LC3A/B Cell Signaling Technologies #12741 for 293 cells, LC3B MilliporeSigma #L7543 for mouse brain), p62 (Cell Signaling Technologies #5114), TFEB (Cell Signaling Technologies #4240 for 293 cells, Bethyl Labs #A303-673 for mouse brain), and LAMP-1 (Developmental Studies Hybridoma Bank #1D4B). The following day, membranes were incubated with IRDye-conjugated secondary antibodies (LiCOR) and scanned on an Odyssey scanner (LiCOR). We utilized antibodies for two detection channels of the Odyssey scanner (IRDye 800CW and IRDye 680LT) to probe blots for multiple proteins. Bands were quantified using ImageStudio Lite software (LiCOR). Representative blots were converted to grayscale for display throughout the manuscript and supplemental information.

### Mouse brain immunostaining

Fixed hemibrains were sliced into 30 μm sections on a sliding microtome, then immunostained as previously described^105^. Sections were incubated overnight with the following primary antibodies: HA tag (Cell Signaling Technologies #3724), SCMAS (Abcam #ab181243), CD68 (Bio-Rad #MCA1957), or Galectin 3 (R&D Systems #AF1197). The following day, sections were incubated with biotinylated species-matched secondary antibodies (Vector Labs) and Vectastain Elite ABC reagent (Vector Labs). Immunolabeling was detected by development with diaminobenzidine (MP Biomedicals).

### Mouse brain imaging and analysis

To analyze the burden of pathology, 10X images of the thalamus were obtained with an EVOS M5000 microscope (ThermoFisher). Images were quantitated with ImageJ^101^ by applying a uniform threshold to determine the area of immunostaining. Slides were coded by animal number to allow the investigator to remain blinded to treatment group until imaging and analysis were complete.

### qPCR

RNA was isolated from cells using RNeasy mini kits (Qiagen) and from tissue using Trizol reagent (ThermoFisher). Samples were treated with DNAse (Qiagen for cells, Invitrogen Turbo DNase for tissue) to remove any genomic DNA, then reverse transcribed with iScript (Bio-Rad). The resulting cDNA was analyzed by qPCR using PowerTrack SYBR Green Master Mix (ThermoFisher) and a QuantStudio3 system (ThermoFisher). PrimeTime pre-designed qPCR primers (Integrated DNA Technologies) were used to detect all transcripts except for *Gapdh*. For HEK-293 cells, *CTSD* (Hs.PT.58.27568031), *LAMP1* (Hs.PT.58.27192505), and *GRN* (Hs.PT.58.2528960.g) were normalized to expression of *HPRT1* (Hs.PT.58v.45621572). For mouse brain, *Ctsd* (Mm.PT.58.7639164), *Gba* (Mm.PT.58.5261591), *Lamp1* (Mm.PT.58.11391099), *Cd63* (Mm.PT.58.31259937), *Atp6v0a1* (Mm.PT.58.8632597), and *Atp6v1a* (Mm.PT.5829802433) were normalized to expression of *Gapdh* (F: GGGAAGCCCATCACCATCTT, R:GCCTTCTCCATGGTGGTGAA).

### Statistics

For all analyses, assumptions of normal distribution were tested by D’Agostino-Pearson test and Anderson-Darling test, and assumptions of equal variance were tested by Brown-Forsythe test and Bartlett’s test using GraphPad Prism 10. Data not meeting these assumptions were log transformed prior to analysis. Cathepsin D levels and LC3 immunoreactivity in *GRN* KO cells were analyzed by *t* test. Nuclear TFEB localization and autophagic flux in *GRN* KO cells were analyzed by two-way ANOVA with factors of genotype and treatment. The GFP/RFP ratio of cells transfected with GFP-LC3-RFP-LC3ΔG was analyzed by restricted maximum likelihood (REML) linear mixed effects model, with factors of *GRN* genotype and treatment (vehicle versus chloroquine). Data from individual cells were blocked by culture, which was included as a random effect in the model. The cumulative frequency distribution of GFP/RFP fluorescence was also analyzed by Kolmogorov-Smirnov test. Progranulin or TFEB correction of autophagic flux in *GRN* KO cells was analyzed by three-way ANOVA with factors of TFEB, genotype, and treatment, followed by two-way ANOVA of the chloroquine-treated cells with factors of progranulin or TFEB and genotype. Due to the expectation that TFEB overexpression would simultaneously increase levels of multiple lysosomal transcripts and proteins, lysosomal transcript expression in *GRN* KO cells and mouse brain, and lysosomal protein levels in mouse brain were analyzed by MANOVA with factors of TFEB and *GRN* genotype (where applicable). TFEB, LC3, and p62 levels in mouse brain were analyzed by *t* test for wild-type mice (Fig. 3) or one-way ANOVA with a factor of treatment for *Grn*^*–/–*^ mice (Fig. 4). Levels of SCMAS, autofluorescence, CD68, and Galectin 3 in *Grn*^*–/–*^ thalamus were analyzed by one-way ANOVA with a factor of treatment. For all ANOVA, MANOVA, or REML, main effects or interactions were followed by either lower-order ANOVA or post-hoc tests, as described in the figure legends. Two-tailed *p* values were calculated in all analyses, with significance defined as *p* < 0.05. ANOVA and *t* tests were conducted with GraphPad Prism 10, MANOVA were conducted with SPSS 27, and REML linear mixed effects model was conducted using the using the lme4^106^ and lmertest^107^ packages in R.

## Electronic supplementary material

Below is the link to the electronic supplementary material.

Supplementary Material 1

## Data Availability

Primary data supporting this manuscript are available from the corresponding author upon request.

## References

[1] Bateman, A. & Bennett, H. P. The granulin gene family: from cancer to dementia. *Bioessays* **31**, 1245–1254. 10.1002/bies.200900086 (2009). 19795409 10.1002/bies.200900086

[2] Cenik, B., Sephton, C. F., Cenik, K., Herz, B., Yu, G. & J. & Progranulin: a proteolytically processed protein at the crossroads of inflammation and neurodegeneration. *J. Biol. Chem.* **287**, 32298–32306. 10.1074/jbc.R112.399170 (2012). 22859297 10.1074/jbc.R112.399170 PMC3463300

[3] Nguyen, A. D., Nguyen, T. A., Martens, L. H., Mitic, L. L. & Farese, R. V. Jr. Progranulin: at the interface of neurodegenerative and metabolic diseases. *Trends Endocrinol. Metab.* **24**, 597–606. 10.1016/j.tem.2013.08.003 (2013). 24035620 10.1016/j.tem.2013.08.003 PMC3842380

[4] Baker, M. et al. Mutations in progranulin cause tau-negative frontotemporal dementia linked to chromosome 17. *Nature* **442**, 916–919 (2006). 16862116 10.1038/nature05016

[5] Cruts, M. et al. Null mutations in progranulin cause ubiquitin-positive frontotemporal dementia linked to chromosome 17q21. *Nature* **442**, 920–924 (2006). 16862115 10.1038/nature05017

[6] Rademakers, R. et al. Common variation in the miR-659 binding-site of GRN is a major risk factor for TDP43-positive frontotemporal dementia. *Hum. Mol. Genet.* **17**, 3631–3642. 10.1093/hmg/ddn257 (2008). 18723524 10.1093/hmg/ddn257 PMC2581433

[7] Bellenguez, C. et al. New insights into the genetic etiology of alzheimer’s disease and related dementias. *Nat. Genet.* **54**, 412–436. 10.1038/s41588-022-01024-z (2022). 35379992 10.1038/s41588-022-01024-z PMC9005347

[8] Nalls, M. A. et al. Identification of novel risk loci, causal insights, and heritable risk for parkinson’s disease: a meta-analysis of genome-wide association studies. *Lancet Neurol.* **18**, 1091–1102. 10.1016/S1474-4422(19)30320-5 (2019). 31701892 10.1016/S1474-4422(19)30320-5 PMC8422160

[9] Hu, F. et al. Sortilin-mediated endocytosis determines levels of the frontotemporal dementia protein, progranulin. *Neuron* **68**, 654–667. 10.1016/j.neuron.2010.09.034 (2010). 21092856 10.1016/j.neuron.2010.09.034 PMC2990962

[10] Zhou, X. et al. Prosaposin facilitates sortilin-independent lysosomal trafficking of progranulin. *J. Cell. Biol.* **210**, 991–1002. 10.1083/jcb.201502029 (2015). 26370502 10.1083/jcb.201502029 PMC4576858

[11] Smith, K. R. et al. Strikingly different clinicopathological phenotypes determined by progranulin-mutation dosage. *Am. J. Hum. Genet.* **90**, 1102–1107. 10.1016/j.ajhg.2012.04.021 (2012). 22608501 10.1016/j.ajhg.2012.04.021 PMC3370276

[12] Almeida, M. R. et al. Portuguese family with the co-occurrence of frontotemporal lobar degeneration and neuronal ceroid lipofuscinosis phenotypes due to progranulin gene mutation. *Neurobiol Aging* 41, 200 e201-205, (2016). 10.1016/j.neurobiolaging.2016.02.019 10.1016/j.neurobiolaging.2016.02.019 27021778

[13] Kamate, M., Detroja, M. & Hattiholi, V. Neuronal ceroid lipofuscinosis type-11 in an adolescent. *Brain Dev.* **41**, 542–545. 10.1016/j.braindev.2019.03.004 (2019). 30922528 10.1016/j.braindev.2019.03.004

[14] Huin, V. et al. Homozygous GRN mutations: new phenotypes and new insights into pathological and molecular mechanisms. *Brain*10.1093/brain/awz377 (2019). 31855245 10.1093/brain/awz377

[15] Holler, C. J., Taylor, G., Deng, Q. & Kukar, T. Intracellular Proteolysis of Progranulin Generates Stable, Lysosomal Granulins that Are Haploinsufficient in Patients with Frontotemporal Dementia Caused by GRN Mutations. *eNeuro* 4, (2017). 10.1523/ENEURO.0100-17.2017 10.1523/ENEURO.0100-17.2017 PMC5562298 28828399

[16] Beel, S. et al. Progranulin functions as a cathepsin D chaperone to stimulate axonal outgrowth in vivo. *Hum. Mol. Genet.*10.1093/hmg/ddx162 (2017). 28453791 10.1093/hmg/ddx162 PMC5886064

[17] Zhou, X. et al. Regulation of cathepsin D activity by the FTLD protein progranulin. *Acta Neuropathol.*10.1007/s00401-017-1719-5 (2017). 28493053 10.1007/s00401-017-1719-5 PMC5568051

[18] Valdez, C. et al. Progranulin-mediated deficiency of cathepsin D results in FTD and NCL-like phenotypes in neurons derived from FTD patients. *Hum. Mol. Genet.*10.1093/hmg/ddx364 (2017). 29036611 10.1093/hmg/ddx364 PMC5886207

[19] Butler, V. J. et al. Progranulin stimulates the in vitro maturation of Pro-Cathepsin D at acidic pH. *J. Mol. Biol.* **431**, 1038–1047. 10.1016/j.jmb.2019.01.027 (2019). 30690031 10.1016/j.jmb.2019.01.027 PMC6613950

[20] Butler, V. J. et al. Multi-Granulin domain peptides bind to Pro-Cathepsin D and stimulate its enzymatic activity more effectively than progranulin in vitro. *Biochemistry* **58**, 2670–2674. 10.1021/acs.biochem.9b00275 (2019). 31099551 10.1021/acs.biochem.9b00275 PMC6666309

[21] Chen, Y. et al. Progranulin associates with hexosaminidase A and ameliorates GM2 ganglioside accumulation and lysosomal storage in Tay-Sachs disease. *J. Mol. Med. (Berl)*. **96**, 1359–1373. 10.1007/s00109-018-1703-0 (2018). 30341570 10.1007/s00109-018-1703-0 PMC6240367

[22] Jian, J. et al. Progranulin recruits HSP70 to beta-Glucocerebrosidase and is therapeutic against gaucher disease. *EBioMedicine* **13**, 212–224. 10.1016/j.ebiom.2016.10.010 (2016). 27789271 10.1016/j.ebiom.2016.10.010 PMC5264254

[23] Arrant, A. E. et al. Impaired beta-glucocerebrosidase activity and processing in frontotemporal dementia due to progranulin mutations. *Acta Neuropathol. Commun.* **7**, 218. 10.1186/s40478-019-0872-6 (2019). 31870439 10.1186/s40478-019-0872-6 PMC6929503

[24] Zhou, X. et al. Progranulin deficiency leads to reduced glucocerebrosidase activity. *PLoS One*. **14**, e0212382. 10.1371/journal.pone.0212382 (2019). 31291241 10.1371/journal.pone.0212382 PMC6619604

[25] Valdez, C., Ysselstein, D., Young, T. J., Zheng, J. & Krainc, D. Progranulin mutations result in impaired processing of prosaposin and reduced glucocerebrosidase activity. *Hum. Mol. Genet.* **29**, 716–726. 10.1093/hmg/ddz229 (2020). 31600775 10.1093/hmg/ddz229 PMC7104673

[26] Schulze, H. & Sandhoff, K. Sphingolipids and lysosomal pathologies. *Biochim. Biophys. Acta*. **1841**, 799–810. 10.1016/j.bbalip.2013.10.015 (2014). 24184515 10.1016/j.bbalip.2013.10.015

[27] Zhou, X. et al. Impaired prosaposin lysosomal trafficking in frontotemporal Lobar degeneration due to progranulin mutations. *Nat. Commun.* **8**, 15277. 10.1038/ncomms15277 (2017). 28541286 10.1038/ncomms15277 PMC5477518

[28] Logan, T. et al. Rescue of a lysosomal storage disorder caused by Grn loss of function with a brain penetrant progranulin biologic. *Cell*10.1016/j.cell.2021.08.002 (2021). 34450028 10.1016/j.cell.2021.08.002 PMC8489356

[29] Ward, M. E. et al. Individuals with progranulin haploinsufficiency exhibit features of neuronal ceroid lipofuscinosis. *Sci. Transl Med.* **9**10.1126/scitranslmed.aah5642 (2017). 10.1126/scitranslmed.aah5642 PMC5526610 28404863

[30] Davis, S. E. et al. Patients with sporadic FTLD exhibit similar increases in lysosomal proteins and storage material as patients with FTD due to GRN mutations. *Acta Neuropathol. Commun.* **11**, 70. 10.1186/s40478-023-01571-4 (2023). 37118844 10.1186/s40478-023-01571-4 PMC10148425

[31] Ahmed, Z. et al. Accelerated lipofuscinosis and ubiquitination in granulin knockout mice suggest a role for progranulin in successful aging. *Am. J. Pathol.* **177**, 311–324. 10.2353/ajpath.2010.090915 (2010). 20522652 10.2353/ajpath.2010.090915 PMC2893674

[32] Filiano, A. J. et al. Dissociation of frontotemporal dementia–related deficits and neuroinflammation in progranulin haploinsufficient mice. *J. Neurosci.* **33**, 5352–5361. 10.1523/JNEUROSCI.6103-11.2013 (2013). 23516300 10.1523/JNEUROSCI.6103-11.2013 PMC3740510

[33] Arrant, A. E., Onyilo, V. C., Unger, D. E. & Roberson, E. D. Progranulin gene therapy improves lysosomal dysfunction and microglial pathology associated with frontotemporal dementia and neuronal ceroid lipofuscinosis. *J. Neurosci.* **38**, 2341. 10.1523/JNEUROSCI.3081-17.2018 (2018). 29378861 10.1523/JNEUROSCI.3081-17.2018 PMC5830520

[34] Hall, N. A., Lake, B. D., Dewji, N. N. & Patrick, A. D. Lysosomal storage of subunit c of mitochondrial ATP synthase in batten’s disease (ceroid-lipofuscinosis). *Biochem. J.* **275** (Pt 1), 269–272 (1991). 1826833 10.1042/bj2750269 PMC1150044

[35] Kominami, E. et al. Specific storage of subunit c of mitochondrial ATP synthase in lysosomes of neuronal ceroid lipofuscinosis (Batten’s disease). *J. Biochem.* **111**, 278–282 (1992). 1533218 10.1093/oxfordjournals.jbchem.a123749

[36] Elleder, M., Sokolová, J. & Hřebíček, M. Follow-up study of subunit c of mitochondrial ATP synthase (SCMAS) in Batten disease and in unrelated lysosomal disorders. *Acta Neuropathol.* **93**, 379–390. 10.1007/s004010050629 (1997). 9113203 10.1007/s004010050629

[37] Nguyen, A. D. et al. Murine knockin model for progranulin-deficient frontotemporal dementia with nonsense-mediated mRNA decay. *Proc. Natl. Acad. Sci. U S A*. **115**, E2849–E2858. 10.1073/pnas.1722344115 (2018). 29511098 10.1073/pnas.1722344115 PMC5866607

[38] Boland, S. et al. Deficiency of the frontotemporal dementia gene GRN results in gangliosidosis. *Nat. Commun.* **13**, 5924. 10.1038/s41467-022-33500-9 (2022). 36207292 10.1038/s41467-022-33500-9 PMC9546883

[39] Götzl, J. K. et al. Common pathobiochemical hallmarks of progranulin-associated frontotemporal Lobar degeneration and neuronal ceroid lipofuscinosis. *Acta Neuropathol.* **127**, 845–860. 10.1007/s00401-014-1262-6 (2014). 24619111 10.1007/s00401-014-1262-6

[40] Robinson, S. et al. Enhanced legumain activity links progranulin deficiency to TDP-43 pathology in frontotemporal lobar degeneration. *bioRxiv*, 2024.2001.2016.575687, (2024). 10.1101/2024.01.16.575687

[41] Smith, D. M. et al. Biochemical, biomarker, and behavioral characterization of the Grn(R493X) mouse model of frontotemporal dementia. *Mol. Neurobiol.* **61**, 9708–9722. 10.1007/s12035-024-04190-9 (2024). 38696065 10.1007/s12035-024-04190-9 PMC11496013

[42] Evers, B. M. et al. Lipidomic and transcriptomic basis of lysosomal dysfunction in progranulin deficiency. *Cell. Rep.* **20**, 2565–2574. 10.1016/j.celrep.2017.08.056 (2017). 28903038 10.1016/j.celrep.2017.08.056 PMC5757843

[43] Tanaka, Y., Matsuwaki, T., Yamanouchi, K. & Nishihara, M. Increased lysosomal biogenesis in activated microglia and exacerbated neuronal damage after traumatic brain injury in progranulin-deficient mice. *Neuroscience* **250**, 8–19. 10.1016/j.neuroscience.2013.06.049 (2013). 23830905 10.1016/j.neuroscience.2013.06.049

[44] Lui, H. et al. Progranulin deficiency promotes Circuit-Specific synaptic pruning by microglia via complement activation. *Cell* **165**, 921–935. 10.1016/j.cell.2016.04.001 (2016). 27114033 10.1016/j.cell.2016.04.001 PMC4860138

[45] Gotzl, J. K. et al. Early lysosomal maturation deficits in microglia triggers enhanced lysosomal activity in other brain cells of progranulin knockout mice. *Mol. Neurodegener*. **13**, 48. 10.1186/s13024-018-0281-5 (2018). 30180904 10.1186/s13024-018-0281-5 PMC6123925

[46] Sardiello, M. et al. A gene network regulating lysosomal biogenesis and function. *Science* **325**, 473–477. 10.1126/science.1174447 (2009). 19556463 10.1126/science.1174447

[47] Settembre, C. et al. TFEB links autophagy to lysosomal biogenesis. *Science* **332**, 1429–1433. 10.1126/science.1204592 (2011). 21617040 10.1126/science.1204592 PMC3638014

[48] Settembre, C. et al. A lysosome-to-nucleus signalling mechanism senses and regulates the lysosome via mTOR and TFEB. *EMBO J.* **31**, 1095–1108. 10.1038/emboj.2012.32 (2012). 22343943 10.1038/emboj.2012.32 PMC3298007

[49] Tanaka, Y. et al. Dysregulation of the progranulin-driven autophagy-lysosomal pathway mediates secretion of the nuclear protein TDP-43. *J. Biol. Chem.* **299**, 105272. 10.1016/j.jbc.2023.105272 (2023). 37739033 10.1016/j.jbc.2023.105272 PMC10641265

[50] Du, H. et al. A multifaceted role of progranulin in regulating amyloid-beta dynamics and responses. *Life Sci. Alliance*. **4**10.26508/lsa.202000874 (2021). 10.26508/lsa.202000874 PMC8200295 34103390

[51] Medina, D. L. et al. Transcriptional activation of lysosomal exocytosis promotes cellular clearance. *Dev. Cell.* **21**, 421–430. 10.1016/j.devcel.2011.07.016 (2011). 21889421 10.1016/j.devcel.2011.07.016 PMC3173716

[52] Spampanato, C. et al. Transcription factor EB (TFEB) is a new therapeutic target for Pompe disease. *EMBO Mol. Med.* **5**, 691–706. 10.1002/emmm.201202176 (2013). 23606558 10.1002/emmm.201202176 PMC3662313

[53] Gatto, F. et al. AAV-mediated transcription factor EB (TFEB) gene delivery ameliorates muscle pathology and function in the murine model of Pompe disease. *Sci. Rep.* **7**, 15089. 10.1038/s41598-017-15352-2 (2017). 29118420 10.1038/s41598-017-15352-2 PMC5678083

[54] Liu, Y. et al. Neuronal-targeted TFEB rescues dysfunction of the autophagy-lysosomal pathway and alleviates ischemic injury in permanent cerebral ischemia. *Autophagy* **15**, 493–509. 10.1080/15548627.2018.1531196 (2019). 30304977 10.1080/15548627.2018.1531196 PMC6351122

[55] Roczniak-Ferguson, A. et al. The transcription factor TFEB links mTORC1 signaling to transcriptional control of lysosome homeostasis. *Sci. Signal.* **5**, ra42. 10.1126/scisignal.2002790 (2012). 22692423 10.1126/scisignal.2002790 PMC3437338

[56] Bauer, D. E., Canver, M. C. & Orkin, S. H. Generation of genomic deletions in mammalian cell lines via CRISPR/Cas9. *J. Vis. Exp.* **e52118**10.3791/52118 (2015). 10.3791/52118 PMC4279820 25549070

[57] Chang, M. C. et al. Progranulin deficiency causes impairment of autophagy and TDP-43 accumulation. *J. Exp. Med.* **214**, 2611–2628. 10.1084/jem.20160999 (2017). 28778989 10.1084/jem.20160999 PMC5584112

[58] Kaizuka, T. et al. An autophagic flux probe that releases an internal control. *Mol. Cell.* **64**, 835–849. 10.1016/j.molcel.2016.09.037 (2016). 27818143 10.1016/j.molcel.2016.09.037

[59] Tanaka, Y., Chambers, J. K., Matsuwaki, T., Yamanouchi, K. & Nishihara, M. Possible involvement of lysosomal dysfunction in pathological changes of the brain in aged progranulin-deficient mice. *Acta Neuropathol. Commun.* **2**, 78. 10.1186/s40478-014-0078-x (2014). 25022663 10.1186/s40478-014-0078-x PMC4149276

[60] Zhang, J. et al. Neurotoxic microglia promote TDP-43 proteinopathy in progranulin deficiency. *Nature*10.1038/s41586-020-2709-7 (2020). 32866962 10.1038/s41586-020-2709-7 PMC7746606

[61] Kashyap, S. N., Boyle, N. R. & Roberson, E. D. Preclinical interventions in mouse models of frontotemporal dementia due to progranulin mutations. *Neurotherapeutics* **20**, 140–153. 10.1007/s13311-023-01348-6 (2023). 36781744 10.1007/s13311-023-01348-6 PMC10119358

[62] Palmieri, M. et al. Characterization of the CLEAR network reveals an integrated control of cellular clearance pathways. *Hum. Mol. Genet.* **20**, 3852–3866. 10.1093/hmg/ddr306 (2011). 21752829 10.1093/hmg/ddr306

[63] Huang, M. et al. Network analysis of the progranulin-deficient mouse brain proteome reveals pathogenic mechanisms shared in human frontotemporal dementia caused by GRN mutations. *Acta Neuropathol. Commun.* **8**, 163. 10.1186/s40478-020-01037-x (2020). 33028409 10.1186/s40478-020-01037-x PMC7541308

[64] Xiao, Q. et al. Enhancing astrocytic lysosome biogenesis facilitates Abeta clearance and attenuates amyloid plaque pathogenesis. *J. Neurosci.* **34**, 9607–9620. 10.1523/JNEUROSCI.3788-13.2014 (2014). 25031402 10.1523/JNEUROSCI.3788-13.2014 PMC4099542

[65] Xiao, Q. et al. Neuronal-Targeted TFEB accelerates lysosomal degradation of APP, reducing Abeta generation and amyloid plaque pathogenesis. *J. Neurosci.* **35**, 12137–12151. 10.1523/JNEUROSCI.0705-15.2015 (2015). 26338325 10.1523/JNEUROSCI.0705-15.2015 PMC4556784

[66] Cheng, L. et al. mTOR-dependent TFEB activation and TFEB overexpression enhance autophagy-lysosome pathway and ameliorate alzheimer’s disease-like pathology in diabetic encephalopathy. *Cell. Commun. Signal.* **21**, 91. 10.1186/s12964-023-01097-1 (2023). 37143104 10.1186/s12964-023-01097-1 PMC10158341

[67] Polito, V. A. et al. Selective clearance of aberrant Tau proteins and rescue of neurotoxicity by transcription factor EB. *EMBO Mol. Med.* **6**, 1142–1160. 10.15252/emmm.201303671 (2014). 25069841 10.15252/emmm.201303671 PMC4197862

[68] Martini-Stoica, H. et al. TFEB enhances astroglial uptake of extracellular Tau species and reduces Tau spreading. *J. Exp. Med.* **215**, 2355–2377. 10.1084/jem.20172158 (2018). 30108137 10.1084/jem.20172158 PMC6122971

[69] Decressac, M. et al. TFEB-mediated autophagy rescues midbrain dopamine neurons from alpha-synuclein toxicity. *Proc. Natl. Acad. Sci. U S A*. **110**, E1817–1826. 10.1073/pnas.1305623110 (2013). 23610405 10.1073/pnas.1305623110 PMC3651458

[70] Arotcarena, M. L. et al. Transcription factor EB overexpression prevents neurodegeneration in experimental synucleinopathies. *JCI Insight*. **4**10.1172/jci.insight.129719 (2019). 10.1172/jci.insight.129719 PMC6777809 31434803

[71] Martini-Stoica, H., Xu, Y., Ballabio, A. & Zheng, H. The Autophagy-Lysosomal pathway in neurodegeneration: A TFEB perspective. *Trends Neurosci.* **39**, 221–234. 10.1016/j.tins.2016.02.002 (2016). 26968346 10.1016/j.tins.2016.02.002 PMC4928589

[72] Cortes, C. J. & La Spada, A. R. TFEB dysregulation as a driver of autophagy dysfunction in neurodegenerative disease: molecular mechanisms, cellular processes, and emerging therapeutic opportunities. *Neurobiol. Dis.* **122**, 83–93. 10.1016/j.nbd.2018.05.012 (2019). 29852219 10.1016/j.nbd.2018.05.012 PMC6291370

[73] Arrant, A. E. et al. Elevated levels of extracellular vesicles in progranulin-deficient mice and FTD-GRN patients. *Ann. Clin. Transl Neurol.*10.1002/acn3.51242 (2020). 33197149 10.1002/acn3.51242 PMC7732244

[74] Yin, F. et al. Exaggerated inflammation, impaired host defense, and neuropathology in progranulin-deficient mice. *J. Exp. Med.* **207**, 117–128. 10.1084/jem.20091568 (2010). 20026663 10.1084/jem.20091568 PMC2812536

[75] Martens, L. H. et al. Progranulin deficiency promotes neuroinflammation and neuron loss following toxin-induced injury. *J. Clin. Invest.* **122**, 3955–3959 (2012). 23041626 10.1172/JCI63113 PMC3484443

[76] Amado, D. A. et al. AAV-Mediated progranulin delivery to a mouse model of progranulin deficiency causes T Cell-Mediated toxicity. *Mol. Ther.* **27**, 465–478. 10.1016/j.ymthe.2018.11.013 (2019). 30559071 10.1016/j.ymthe.2018.11.013 PMC6369714

[77] Kim, J. Y., Grunke, S. D., Levites, Y., Golde, T. E. & Jankowsky, J. L. Intracerebroventricular viral injection of the neonatal mouse brain for persistent and widespread neuronal transduction. *J. Vis. Exp.* **51863**10.3791/51863 (2014). 10.3791/51863 PMC4199253 25286085

[78] Root, J. et al. Granulins rescue inflammation, lysosome dysfunction, and neuropathology in a mouse model of progranulin deficiency. *BioRxiv* **2023.2004.2017.536004**10.1101/2023.04.17.536004 (2023). 10.1016/j.celrep.2024.114985 PMC11773623 39565694

[79] Skibinski, G. et al. Mutations in the endosomal ESCRTIII-complex subunit CHMP2B in frontotemporal dementia. *Nat. Genet.* **37**, 806–808 (2005). 16041373 10.1038/ng1609

[80] Watts, G. D. et al. Inclusion body myopathy associated with Paget disease of bone and frontotemporal dementia is caused by mutant valosin-containing protein. *Nat. Genet.* **36**, 377–381 (2004). 15034582 10.1038/ng1332

[81] Freischmidt, A. et al. Haploinsufficiency of TBK1 causes Familial ALS and fronto-temporal dementia. *Nat. Neurosci.* **18**, 631–636. 10.1038/nn.4000 (2015). 25803835 10.1038/nn.4000

[82] Gijselinck, I. et al. Loss of TBK1 is a frequent cause of frontotemporal dementia in a Belgian cohort. *Neurology* **85**, 2116–2125. 10.1212/WNL.0000000000002220 (2015). 26581300 10.1212/WNL.0000000000002220 PMC4691687

[83] Pottier, C. et al. Whole-genome sequencing reveals important role for TBK1 and OPTN mutations in frontotemporal Lobar degeneration without motor neuron disease. *Acta Neuropathol.* **130**, 77–92. 10.1007/s00401-015-1436-x (2015). 25943890 10.1007/s00401-015-1436-x PMC4470809

[84] DeJesus-Hernandez, M. et al. Expanded GGGGCC hexanucleotide repeat in noncoding region of C9ORF72 causes chromosome 9p-linked FTD and ALS. *Neuron* **72**, 245–256. 10.1016/j.neuron.2011.09.011 (2011). doi:S0896-6273(11)00828-2 [pii]. 21944778 10.1016/j.neuron.2011.09.011 PMC3202986

[85] Renton, A. E. et al. A hexanucleotide repeat expansion in C9ORF72 is the cause of chromosome 9p21-linked ALS-FTD. *Neuron* **72**, 257–268. 10.1016/j.neuron.2011.09.010 (2011). doi:S0896-6273(11)00797-5 [pii]. 21944779 10.1016/j.neuron.2011.09.010 PMC3200438

[86] Clayton, E. L. et al. Frontotemporal dementia caused by CHMP2B mutation is characterised by neuronal lysosomal storage pathology. *Acta Neuropathol.* **130**, 511–523. 10.1007/s00401-015-1475-3 (2015). 26358247 10.1007/s00401-015-1475-3 PMC4575387

[87] Beckers, J., Tharkeshwar, A. K. & Van Damme, P. C9orf72 ALS-FTD: recent evidence for dysregulation of the autophagy-lysosome pathway at multiple levels. *Autophagy* **17**, 3306–3322. 10.1080/15548627.2021.1872189 (2021). 33632058 10.1080/15548627.2021.1872189 PMC8632097

[88] Wani, A. et al. Neuronal VCP loss of function recapitulates FTLD-TDP pathology. *Cell. Rep.* **36**, 109399. 10.1016/j.celrep.2021.109399 (2021). 34289347 10.1016/j.celrep.2021.109399 PMC8383344

[89] Piras, A., Collin, L., Gruninger, F., Graff, C. & Ronnback, A. Autophagic and lysosomal defects in human tauopathies: analysis of post-mortem brain from patients with Familial alzheimer disease, corticobasal degeneration and progressive supranuclear palsy. *Acta Neuropathol. Commun.* **4**, 22. 10.1186/s40478-016-0292-9 (2016). 26936765 10.1186/s40478-016-0292-9 PMC4774096

[90] Root, J., Merino, P., Nuckols, A., Johnson, M. & Kukar, T. Lysosome dysfunction as a cause of neurodegenerative diseases: lessons from frontotemporal dementia and amyotrophic lateral sclerosis. *Neurobiol. Dis.* **154**, 105360. 10.1016/j.nbd.2021.105360 (2021). 33812000 10.1016/j.nbd.2021.105360 PMC8113138

[91] Todd, T. W., Shao, W., Zhang, Y. J. & Petrucelli, L. The endolysosomal pathway and ALS/FTD. *Trends Neurosci.* **46**, 1025–1041. 10.1016/j.tins.2023.09.004 (2023). 37827960 10.1016/j.tins.2023.09.004 PMC10841821

[92] Marsan, E. et al. Astroglial toxicity promotes synaptic degeneration in the thalamocortical circuit in frontotemporal dementia with GRN mutations. *J. Clin. Invest.* **133**10.1172/JCI164919 (2023). 10.1172/JCI164919 PMC10014110 36602862

[93] Wu, Y. et al. Microglial lysosome dysfunction contributes to white matter pathology and TDP-43 proteinopathy in GRN-associated FTD. *Cell. Rep.* **36**, 109581. 10.1016/j.celrep.2021.109581 (2021). 34433069 10.1016/j.celrep.2021.109581 PMC8491969

[94] Krabbe, G. et al. Microglial NFkappaB-TNFalpha hyperactivation induces obsessive-compulsive behavior in mouse models of progranulin-deficient frontotemporal dementia. *Proc. Natl. Acad. Sci. U S A*. **114**, 5029–5034. 10.1073/pnas.1700477114 (2017). 28438992 10.1073/pnas.1700477114 PMC5441749

[95] Feiler, M. S. et al. TDP-43 is intercellularly transmitted across axon terminals. *J. Cell. Biol.* **211**, 897–911. 10.1083/jcb.201504057 (2015). 26598621 10.1083/jcb.201504057 PMC4657165

[96] Iguchi, Y. et al. Exosome secretion is a key pathway for clearance of pathological TDP-43. *Brain* **139**, 3187–3201. 10.1093/brain/aww237 (2016). 27679482 10.1093/brain/aww237 PMC5840881

[97] Ran, F. A. et al. Genome engineering using the CRISPR-Cas9 system. *Nat. Protoc.* **8**, 2281–2308. 10.1038/nprot.2013.143 (2013). 24157548 10.1038/nprot.2013.143 PMC3969860

[98] Konermann, S. et al. Optical control of mammalian endogenous transcription and epigenetic States. *Nature* **500**, 472–476. 10.1038/nature12466 (2013). 23877069 10.1038/nature12466 PMC3856241

[99] Savell, K. E. et al. A Neuron-Optimized CRISPR/dCas9 Activation System for Robust and Specific Gene Regulation. *eNeuro* 6, ENEURO.0495 – 0418., (2019). 10.1523/ENEURO.0495-18.2019 10.1523/ENEURO.0495-18.2019 PMC6412672 30863790

[100] Stirling, D. R. et al. CellProfiler 4: improvements in speed, utility and usability. *BMC Bioinform.* **22**, 433. 10.1186/s12859-021-04344-9 (2021). 10.1186/s12859-021-04344-9 PMC8431850 34507520

[101] Schindelin, J. et al. Fiji: an open-source platform for biological-image analysis. *Nat. Methods*. **9**, 676–682. 10.1038/nmeth.2019 (2012). 22743772 10.1038/nmeth.2019 PMC3855844

[102] St Martin, J. L. et al. Dopaminergic neuron loss and up-regulation of chaperone protein mRNA induced by targeted over-expression of alpha-synuclein in mouse substantia Nigra. *J. Neurochem*. **100**, 1449–1457. 10.1111/j.1471-4159.2006.04310.x (2007). 17241127 10.1111/j.1471-4159.2006.04310.x

[103] Percie du Sert. The ARRIVE guidelines 2.0: updated guidelines for reporting animal research. *PLoS Biol.* **18**, e3000410. 10.1371/journal.pbio.3000410 (2020). 32663219 10.1371/journal.pbio.3000410 PMC7360023

[104] Arrant, A. E., Filiano, A. J., Unger, D. E., Young, A. H. & Roberson, E. D. Restoring neuronal progranulin reverses deficits in a mouse model of frontotemporal dementia. *Brain* **140**, 1447–1465. 10.1093/brain/awx060 (2017). 28379303 10.1093/brain/awx060 PMC5965303

[105] Palop, J. J., Mucke, L. & Roberson, E. D. Quantifying biomarkers of cognitive dysfunction and neuronal network hyperexcitability in mouse models of alzheimer’s disease: depletion of calcium-dependent proteins and inhibitory hippocampal remodeling. *Methods Mol. Biol.* **670**, 245–262 (2011). 20967595 10.1007/978-1-60761-744-0_17 PMC8153735

[106] Bates, D., Mächler, M., Bolker, B. & Walker, S. Fitting linear Mixed-Effects models using lme4. *J. Stat. Softw.* **67**, 1–48. 10.18637/jss.v067.i01 (2015).

[107] Kuznetsova, A., Brockhoff, P. B. & Christensen, R. H. B. LmerTest package: tests in linear mixed effects models. *J. Stat. Softw.* **82**, 1–26. 10.18637/jss.v082.i13 (2017).

[108] Lord, S. J., Velle, K. B., Mullins, R. D. & Fritz-Laylin, L. K. SuperPlots: communicating reproducibility and variability in cell biology. *J. Cell. Biol.* **219**10.1083/jcb.202001064 (2020). 10.1083/jcb.202001064 PMC7265319 32346721

