# Supplementary material for: TFEB overexpression alleviates autophagy-lysosomal deficits caused by progranulin insufficiency
Source: Sci Rep. 2025 Jul 19;15:26217. doi: 10.1038/s41598-025-12268-0 (PMC12276339; doi:10.1038/s41598-025-12268-0)
Supplement: Supplementary file 1 — Supplementary Material 1 [file 41598_2025_12268_MOESM1_ESM.pdf]

Supplementary Information for: TFEB overexpression alleviates autophagy-lysosomal deficits caused by progranulin insufficiency

Wren O. Nader, Kaylan S. Brown, Nicholas R. Boyle, Azariah K. Kaplelach, Shaimaa M. Abdelaziz, Skylar E. Davis, Qays Aljabi, Ahmad R. Hakim, Amelia G. Davidson, Giacynta A. Vollmer, Leah C. Wright, J. Bailey Echols, Joelle Saad, Nicholas S. Pena, and Andrew E. Arrant

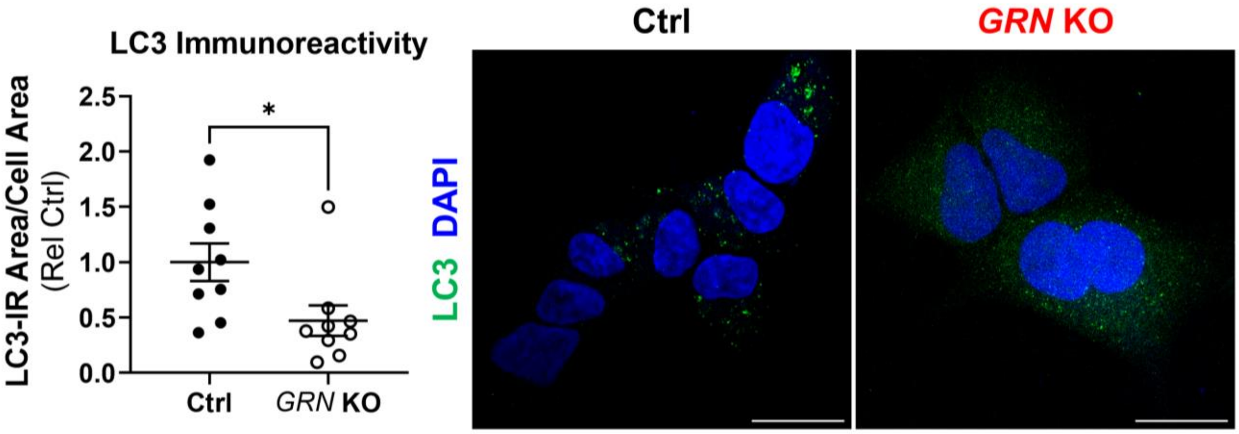

Figure S1 – Fewer LC3-Immunoreactive Puncta in *GRN* KO Cells.
Control and *GRN* KO cells were immunostained for LC3, then imaged at 60X on a confocal microscope. *GRN* KO cells had fewer LC3-immunoreactive puncta than control cells (*t* test, p = 0.0121, n = 9 replicates from 3 independent cultures). The area of LC3-immunoreactive puncta was corrected for cell density in each field of view based on the area of DAPI labeling. *GRN* KO cells also appeared to have a more diffuse, faint pattern of LC3 immunostaining than controls. Scale bars represent 20 μm.

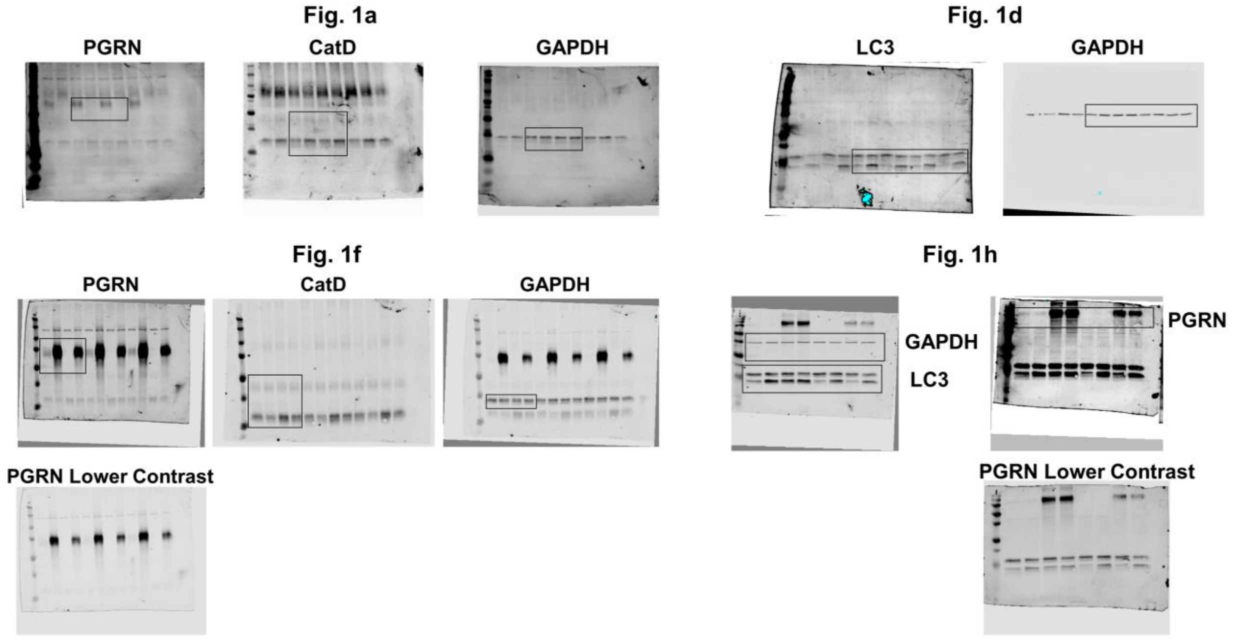

Figure S2 – Full-blot images used for Figure 1. Images are grouped by the corresponding panel in figure 1 from the main text. Images were converted to grayscale from the green or red channels of original scan files. In some instances, blot images were digitally rotated to straighten bands for cropping. The approximate area cropped for the main figure is shown with a box on each blot. Progranulin blots in 1f and 1h are shown at high contrast in the main figure to enable visualization of endogenous progranulin from control cells alongside the high levels of progranulin expressed in cells transfected with the progranulin plasmid. Lower contrast images of these blots are shown below the images used in the main figures. Samples used for Fig. 1a and 1f were run on 10% polyacrylamide gels, while those used for LC3 analysis in Fig. 1d and 1h were run on 15% polyacrylamide gels. PGRN = progranulin, CatD = cathepsin D.

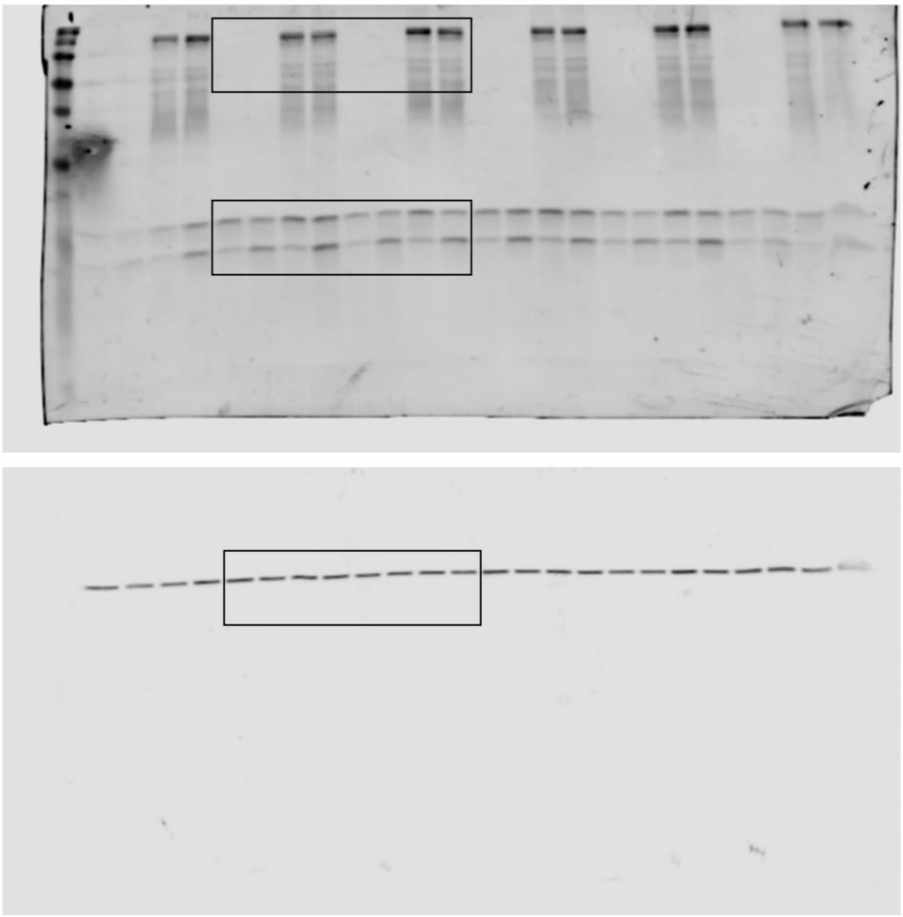

Figure S3 – Full-blot images used for Figure 2. Both images were obtained from the same blot using red (TFEB, LC3) and green (GAPDH) channels. The approximate area cropped for the main figure is shown with a box on each blot. Samples were run on a 15% polyacrylamide gel. The TFEB band occurs at a higher molecular weight than endogenous TFEB because the plasmid used for these experiments expressed a TFEB-GFP fusion protein.

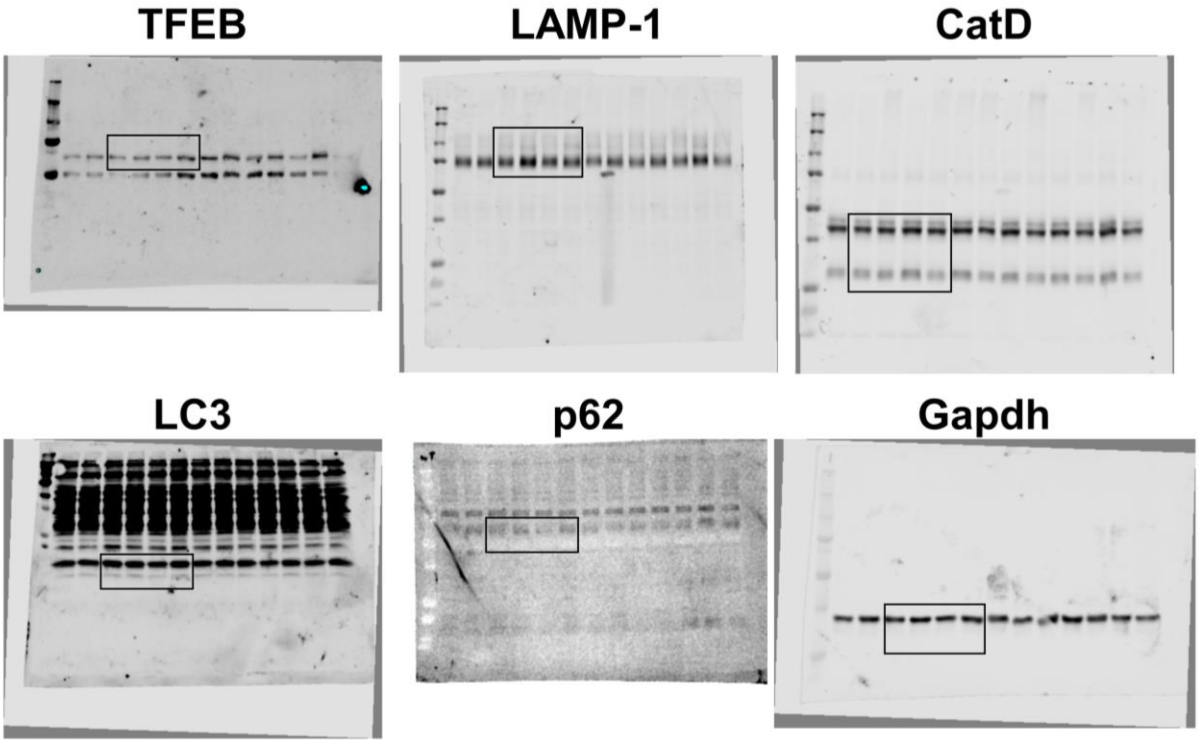

Figure S4 – Full-blot images used for Figure 3. The approximate area cropped for the main figure is shown with a box on each blot. In some instances, blot images were digitally rotated to straighten bands for cropping. All blots except for LC3 were obtained from samples run on 10% polyacrylamide gels. The LC3 blot was obtained from samples run on a 15% polyacrylamide gel. CatD = cathepsin D.

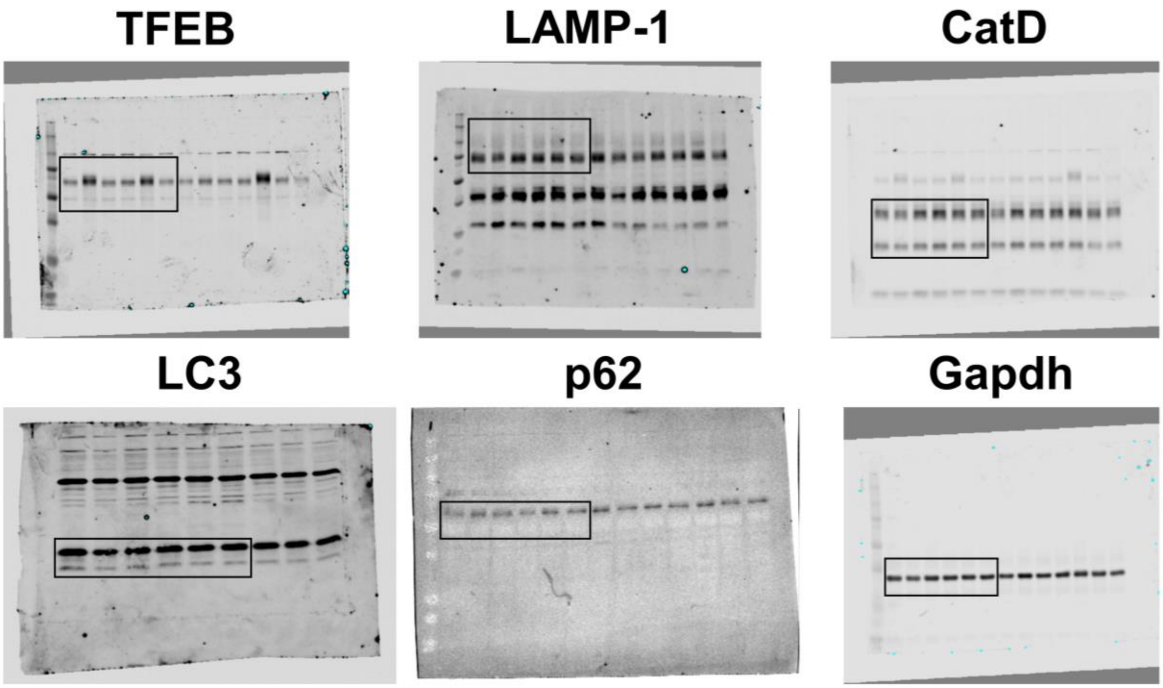

Figure S5 – Full-blot images used for Figure 4. The approximate area cropped for the main figure is shown with a box on each blot. In some instances, blot images were digitally rotated to straighten bands for cropping. All blots except for LC3 were obtained from samples run on 10% polyacrylamide gels. The LC3 blot was obtained from samples run on a 15% polyacrylamide gel. CatD = cathepsin D.

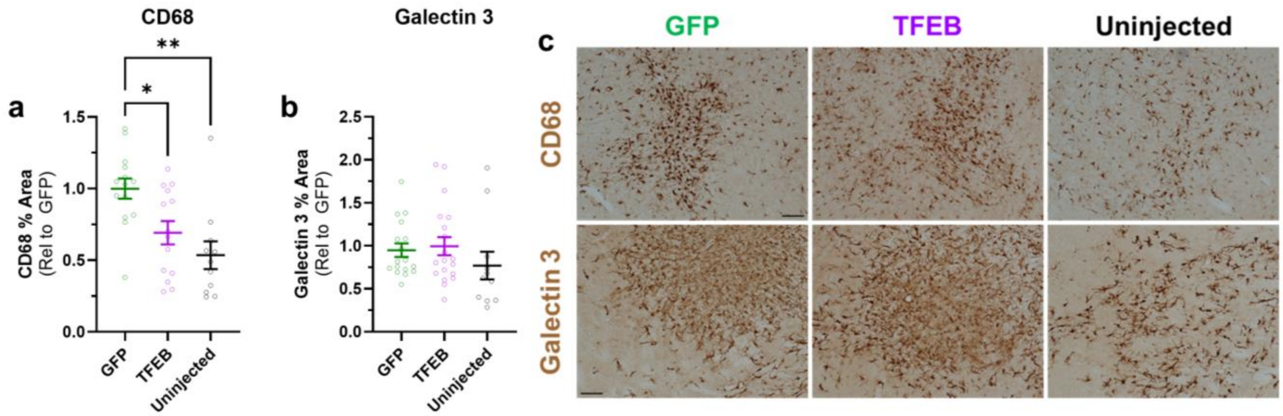

Figure S6 – Increased CD68 at the AAV Injection Site. a, AAV-GFP increased immunoreactivity for CD68, a marker for reactive microglia and monocytes, at the injection site in the VPM/VPL thalamus (ANOVA effect of treatment, p = 0.0013, * = p = 0.0412, ** = p = 0.0010 by Tukey's post-hoc test, n = 11–14/group). While AAV-TFEB–treated mice had lower CD68 immunoreactivity than AAV-GFP–treated mice, many mice exhibited abnormal patterns of CD68 at the injection site as well. **b**, A similar trend was observed with Galectin 3 immunostaining, which labels reactive microglia in *Grn*$^{-/-}$ mice, though this did not reach statistical significance (ANOVA effect of treatment, p = 0.0962, n = 11–19 mice/group) and both AAV-GFP and AAV-TFEB–treated mice trended higher than uninjected mice. Representative 10X images are shown in **c** with 100 μm scale bars.
